# Scaling-up of carbon dots hydrothermal synthesis from sugars in a continuous flow microreactor system for biomedical application as *in vitro* antimicrobial drug nanocarrier

**DOI:** 10.1080/14686996.2023.2260298

**Published:** 2023-10-17

**Authors:** Siriboon Supajaruwong, Sirawich Porahong, Agung Wibowo, Yu-Sheng Yu, Mohd Jahir Khan, Pisut Pongchaikul, Pattaraporn Posoknistakul, Navadol Laosiripojana, Kevin C.-W. Wu, Chularat Sakdaronnarong

**Affiliations:** a Department of Chemical Engineering, Faculty of Engineering, Mahidol University, Nakhon Pathom, Thailand; b Department of Chemical Engineering, National Taiwan University, Taipei, Taiwan; c Chakri Naruebodindra Medical Institute, Faculty of Medicine Ramathibodi Hospital, Mahidol University, Samut Prakarn, Thailand; d The Joint Graduate School of Energy and Environment, King Mongkut’s University of Technology Thonburi, Bangkok, Thailand; e National Health Research Institutes, Institute of Biomedical Engineering and Nanomedicine, Miaoli, Taiwan

**Keywords:** Carbon dots, continuous hydrothermal method, drug nanocarrier, antibacterial activity, L-929 cell cytotoxicity, health and well-being

## Abstract

Carbon dots (CDs) are a new class of nanomaterials exhibiting high biocompatibility, water solubility, functionality, and tunable fluorescence (FL) property. Due to the limitations of batch hydrothermal synthesis in terms of low CDs yield and long synthesis duration, this work aimed to increase its production capacity through a continuous flow reactor system. The influence of temperature and time was first studied in a batch reactor for glucose, xylose, sucrose and table sugar precursors. CDs synthesized from sucrose precursor exhibited the highest quantum yield (QY) (175.48%) and the average diameter less than 10 nm (~6.8 ± 1.1 nm) when synthesized at 220°C for 9 h. For a flow reactor system, the best condition for CDs production from sucrose was 1 mL min^−1^ flow rate at 280°C, and 0.2 MPa pressure yielding 53.03% QY and ~ 6.5 ± 0.6 nm average diameter (6.6 mg min^−1^ of CDs productivity). CDs were successfully used as ciprofloxacin (CP) nanocarrier for antimicrobial activity study. The cytotoxicity study showed that no effect of CDs on viability of L-929 fibroblast cells was detected until 1000 µg mL^−1^ CDs concentration. This finding demonstrates that CDs synthesized via a flow reactor system have a high zeta potential and suitable surface properties for nano-theranostic applications.

## IMPACT STATEMENT

In this work, we focused on a scaled-up production of CDs from various sugar monomers from a batch hydrothermal synthesis to a continuous flow microreactor system. From the result, the novelties of the work are as follows:

- Successful CDs’ production with high capacity of 6.6 mg CDs min^−1^ was achieved from sucrose in a continuous system at the reaction time of only 4.4 min at moderate temperature (280–350°C) and pressure (<2 MPa).
- The developed process was greatly superior than the process in a batch reactor which provided only 0.19 mg CDs min^−1^ when the reaction time was 9 or 12 h.
- The produced CDs were subsequently used as *in vitro* antimicrobial drug nanocarrier due to its high zeta potential indicating its high stability in aqueous solution, thus led to enhanced CP loading capacity.
- The pH-controlled released of CP from CP@CDs conjugates worked efficiently toward gram-positive and gram-negative bacterial strains, namely, *Staphylococcus aureus, Escherichia coli* and *Pseudomonas aeruginosa*.
- *In vitro* cytotoxicity against mammalian fibroblast cells (L-929 cells) was also carried on. The findings demonstrated that CDs potentially serve as a new type of nanocarrier with good functionality, FL properties and markedly high biocompatibility for further nanomedicine and nanotherapeutic.
- Their utilization is potentially combined with photonic property such as bioimaging for rapid *in vivo* monitoring of targeted intracellular drug-release toward specific cells, sensitive neurodegeneration detection as well as photo-responsive anti-inflammatory wound healing application.

## GRAPHICAL ABSTRACT

**Figure:**
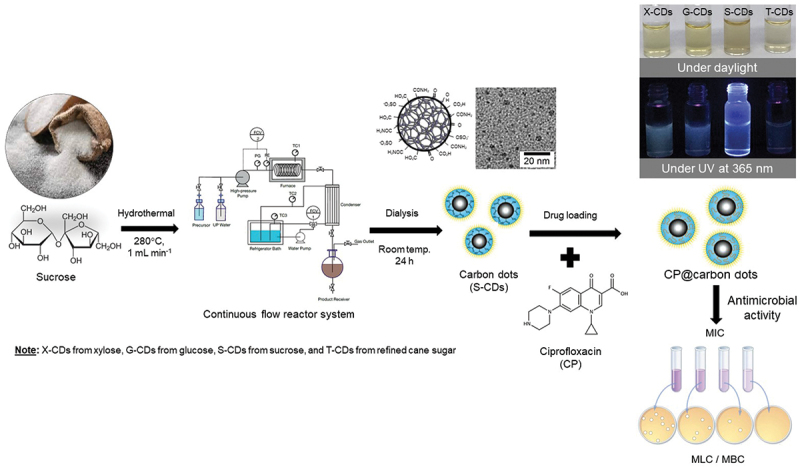

## 1. Introduction

Carbon dots (CDs) are a class of nanostructured carbon materials that has gained popularity recently. CDs can be synthesized from biological carbon-containing materials and, thus, exhibit low toxicity and good solubility. CDs also have excellent and tunable fluorescence (FL) properties along with a good optical stability [1]. In addition, the surface chemistry of CDs can be easily modified to the surface structure specific for special applications in various areas, e.g. electronics, photocatalysis and FL photonics. Due to the above properties, CDs are used in a wide range of applications such as detection of ions [2], catalysts for selective reaction [3] and energy storage [4]. CDs exhibit remarkable biocompatibility in comparison with metal-based nanomaterials, thus extensively used in nanomedicine [5] such as drug nano-carrier [6], bioimaging agent [7], biosensor [8] and the diagnostic substance of various diseases. Sugars, especially glucose from cellulose and starch and sucrose from sugarcane, are interesting carbon sources and potential precursors for CDs production.

According to the literature, conventional CDs synthesis methods were problematic owing to the small quantity of CDs produced and long synthesis time for the batch reaction [9–11], and thus the CDs productivity is insufficient for the commercialization demand. Therefore, a continuous CDs production system was designed based on engineering principles to maintain the efficacy of the reaction while the production is expanded to continuous system [12]. High temperature and pressure are necessary for maintaining the carbonization reaction in which carbon-containing molecules undergo dehydration, and condensation reaction to eliminate oxygen and hydrogen atoms from the precursor [13,14]. Consequently, the majority of carbon molecules in precursors were formed into CDs structure. CDs production has currently been studied in microreactors [15,16], high temperature and pressurized reactors [13]. It has been reported that microfluidic reactors [16] can produce CDs with high quantum yield (QY) (60.1%) in <5 min time) at 160°C from citric acid, ethylenediamine and water. The most suitable tube configuration was linear-like relative to double-snake-like and snake-like microreactors. It was well known that the addition of amine into CDs (nitrogen-doped CDs) synthesis provided CDs with stronger FL; however, no mass yield of CDs and conversion of carbon recovery from the precursor to CDs product were revealed. Another work reported a continuous CDs production using microreactor from ascorbic acid in dimethyl sulfoxide solvent at 180–240°C, nevertheless low productivity of 10–80 µL min^−1^, and low QY at 2.6% were obtained [17]. Later, CDs synthesis from citric acid (CA) and urea was studied in microreactor at flow rate range from 2.2 × 10^−3^ to 3.64 × 10^−3^ mol min^−1^ of citric acid and 0.36 × 10^−3^ to 1.80 × 10^−3^ mol min^−1^ of urea at the ratios of CA to urea were 10:1, 3:1 and 1.5:1. However, only the broaden emission range of CDs from blue to red color was targeted and no QY was reported [15]. Recently, CDs production from glucose in a continuous reactor system at 450°C and 24.8 MPa using supercritical water condition at the reaction time of ca. 2 min was reported. Although it was the first time to report the conversion of glucose to CDs in a continuous system with a high mass yield of 7.7 mg min^−1^, however a very low QY of as-prepared CDs from glucose was obtained at only 0.3% QY while nitrogen-doped CDs provided 15% QY from the same system computed in a relative to QY of quinone sulfate in 0.1 M H_2_SO_4_ [13]. Since the superheated steam at extremely high temperature and pressure were required in this research, the cost of investment and operation cost are relatively high and may not be feasible for a scaled-up process.

Consequently, in this work, we designed and developed a flow reactor system equipped with moderate pressurized liquid chromatography pump with lower range of temperature less than 350°C for CDs synthesis from a continuously fed sugar solution into the conventional heating furnace. Variation of reaction temperature and time by adjusting the flow rate of sugar precursor to produce CDs with high QY and high mass yield was investigated. Analysis of main by products and intermediates from the reaction as well as carbon recovery was reported. The properties of generated CDs from a continuous system were compared with those synthesized from a batch hydrothermal synthesis method. The CDs characterization and functionalization for the subsequent study on antimicrobial drug delivery using CDs as nano-carrier agent along with its toxicity toward various cell lines were discussed herein.

## 2. Experimental details

### 2.1. Materials

D(+)-xylose was analytical grade and purchased from Daejung, Korea. D(+)-glucose monohydrate was analytical grade and purchased from Sigma-Aldrich Inc (Germany). D(+)-sucrose was analytical grade and purchased from Carlo Erba, France. Table sugar was commercial grade refine cane sugar (Mitrphol, Thailand). Ciprofloxacin (CP) 98% (Pharmacopoeia grade) was purchased from Fisher Scientific, USA. Ultrapure water was produced by a water purification system (New Human UP System, Korea) with 18.2 MΩ.cm resistivity at 25°C. Sulfuric acid (HPLC grade) was purchased from Merck, Germany. Quinine sulfate dihydrate (99+%) was purchased from Acros Organics, Belgium. All the chemicals were obtained from commercial sources and used without modification.

For MTT assay of CDs, minimal essential medium alpha (MEM-Alpha; nucleosides, powder), Dulbecco’s modified Eagle’s medium (DMEM; high glucose, pyruvate, powder), fetal bovine serum (FBS) and antibiotic-antimycotic (anti-anti) were purchased from Gibco (Billings, Montana, U.S.A.). Phosphate-buffered saline (PBS 10×) was obtained from Omics Bio (New Taipei City, Taiwan). Sodium bicarbonate, dimethyl sulfoxide (DMSO) and 3-(4,5-dimethylthiazol-2-yl)-2,5-diphenyl tetrazolium bromide (MTT) were purchased from Sigma-Aldrich Inc (Darmstadt, Germany). L-929 cell line was obtained from the National Health Research Institutes (Miaoli, Taiwan).

### 2.2. Synthesis of CDs by a batch hydrothermal method

The CDs were synthesized by a batch hydrothermal method from xylose, glucose, sucrose and table sugar. First, 4 g sugar was dissolved in 80 mL of ultrapure water (5%w/v). Then, the sugar solution was added into a Teflon-lined stainless-steel autoclave and heated at 200°C or 220°C for 6, 9 and 12 h. After the reaction ended, the solution was cooled down to the room temperature naturally. A dark brown solution containing CDs was separated from solid residue using centrifugation at 11,000 rpm for 20 min. Afterwards, the solution was subjected to microfiltration with a 0.22-μm filter membrane. The liquid aliquot was taken for high-performance liquid chromatography (HPLC) to quantify residual sugar, organic acids and furan derivatives generated during hydrothermal reaction. The CDs solution was further purified using dialysis membranes (MWCO 1.0 kDa, Spectra/Por®) for 48 h to eliminate impurities, *e.g.* furan byproducts, remaining sugars and organic acids [18–21].

### 2.3. Synthesis of CDs by a continuous hydrothermal method in a flow reactor system

**Figure 1.**
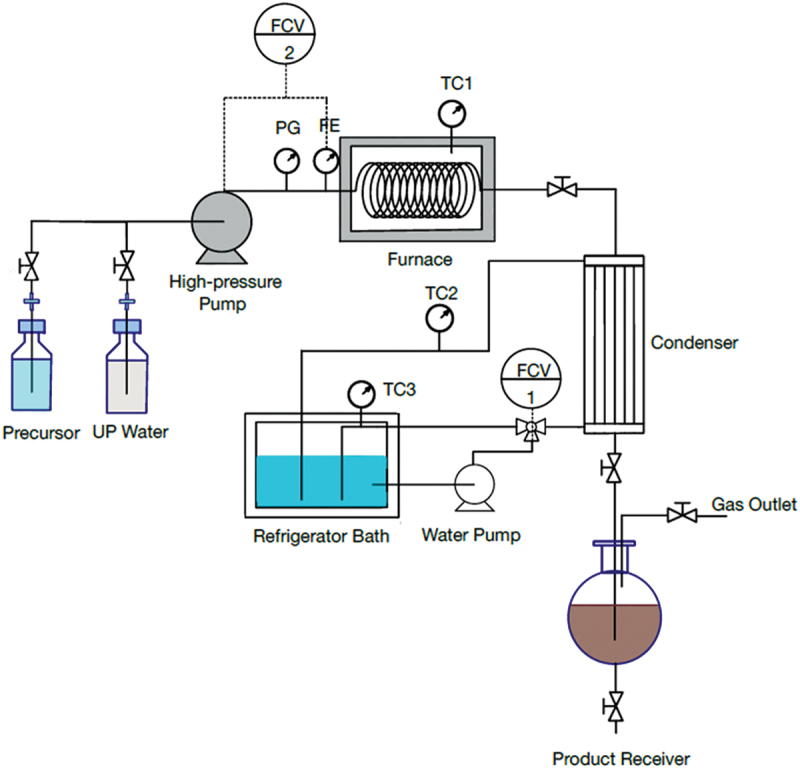
Schematic diagram for CDs synthesis by a continuous hydrothermal method in a pressurized flow reactor system.

The designed system for a continuous hydrothermal synthesis of CDs from sugar solution is shown in Figure 1. The equipment was divided into three parts: 1) a feeding section containing feed reservoir and a high-pressure pump, 2) a reaction section containing a stainless-steel flow reactor and a furnace, and 3) a condensation section containing a condenser system and a product receiver. In this study, sucrose (5%w/v) was selected from the previous experiment as the precursor for the continuous CDs production. A freshly prepared sucrose solution was stirred for completely mixing and then degassed by ultrasonic generator (CPX1800H-E, China) at room temperature (25°C). Before starting the injection of sucrose solution into the flow reactor, the high-pressure pump was purged with ultrapure water to eliminate the gas and clean up impurities in tube. After that the sucrose solution was injected by the high-pressure pump at a fixed flow rate (1, 5 and 10 mL min^−1^) into the feed section through a stainless-steel tube (0.75 mm inner diameter or 750 µm, and 10 m length) into the reaction zone. The reaction zone consists of a furnace (heating rate of 5°C min^−1^) in which the reaction temperature between 240 and 350°C was investigated at the pressure ranged from 0.2 to 1.8 MPa. The product after reaction passed through the condenser zone and the CDs sample was started to collect at the steady-state condition at ca. five times of residence time for each flow rate. The CDs solution was subjected to microfiltration with a 0.22-μm filter membrane, and the liquid aliquot was taken for HPLC analysis to quantify residual sugar, organic acids and furan derivatives. The CDs sample was dialyzed using Spectra/Por® Dialysis membrane (MWCO 1.0 kDa) for 48 h prior to analysis and characterization.

### 2.4. Characterization of CDs

The liquid samples after hydrothermal reaction of sugars were analyzed using HPLC (Waters Alliance 2690 model, U.S.A.), with a refractive index (RI) detector. The analysis was performed using a Bio-Rad Aminex HPX-87 H ion exclusion column (300 × 7.8 mm) to detect sugars, organic acids and other intermediate compounds. The mobile phase used for this analysis was 0.005 M H_2_SO_4_, with a flow rate of 0.5 mL min^−1^. For intermediate quantification, liquid chromatography/mass spectroscopy (LC/MS/MS Orbitrap technique) using Orbitrap HF mass spectroscopy (Thermo Scientific™, USA) with electrospray ion source operated at 3.2 kV. The intermediate product was dried and dissolved in 0.1% formic acid in solubilization solution (50% methanol/50% Water). For analysis, 5 µL of prepared sample was injected into an analytical column (2.1 μm HPLC Column, Ascentis® Express C18), and column temperature was controlled at 55°C when the mobile phase was composed of 0.1% formic acid in water and 0.1% formic acid in acetonitrile. For MS spectral data analysis, the most abundant precursor ions from the survey scan (50–650 m/z) were selected for high-energy collisional dissociation fragmentation. %Carbon recovery was calculated by a ratio of total amount of carbon atom in products (all acids and furan derivatives as well as CDs) based on total amount of carbon atom in sugar precursor. The FL emission characteristics of CDs were measured by varying an excitation wavelength between 240 and 400 nm. FL intensity of CDs was recorded by a spectrofluorometer (Model FP-6200, Perkin Elmer, Japan). The UV-Vis spectroscopy was performed to analyze UV-vis absorption of the synthesized CDs using a double-beam spectrophotometer (Model UV-1800, Thermo Fisher Scientific, Japan) in a wavelength range between 200 and 700 nm. The QY of the CDs was calculated using Equation (1) when the reference was quinine sulfate (QY = 54%) dissolved in 0.1 M H_2_SO_4_. Dilution factor from the original CDs solution was additionally applied to correct the %QY value.

(1) $$QY_{CDs}=QY_{Ref}\;\times \frac{I_{CDs}}{I_{Ref}}\;\times \;\frac{A_{Ref}}{A_{CDs}}\;\times \;\frac{\eta_{CDs}^{2}}{\eta_{Ref}^{2}}$$

where I is the integrated peak area of the highest FL spectrum at the specific excitation wavelength (λ_Ex_), A is the UV-vis absorbance at the specific wavelength of λ_Ex_ aforementioned, and η is the refractive index of the solvents [22]. X-ray photoelectron spectroscopy (XPS) was used to elucidate the surface elemental composition and structure of CD samples (Model PHI5000 Versa Probe II, ULVAC-PHI Inc., Japan), and the XPS spectra were fitted by PHI MultiPak XPS software. The crystal plane analysis of the CDs samples was analyzed by X-ray diffractometry (XRD) (Model D2 PHASER, BRUKER, Germany), and Raman spectroscopy (XPloRA Plus, Horiba, France). Zeta potential measurement was done in triplicates for each sample using a nanoPartica SZ-100V2 series analyzer (Malvern Panalytical, Japan). The CDs size and morphology were analyzed by high-resolution transmission electron microscope (TEM) at 200 kV (JEM-2100 Plus, JEOL, Japan).

### 2.5. Study on loading capacity of ciprofloxacin onto CDs (CP@CDs)

For the synthesis of CP@CDs, 2 mL of CP solution at a concentration of 100, 200 or 400 µmol L^−1^ was added to 8 mL of CDs solution (2 mg mL^−1^), and the mixture was stirred for 24 h at 25°C, 100 rpm. Then, the CP@CDs solution was dialyzed using dialysis membrane (MWCO 1.0 kDa) for 8 h to remove free CP. Loading amount and drug loading efficiency (DLE) of CP on CDs were calculated using Equation (2) and Equation (3), respectively, when the concentration of CP in the solution was quantified by UV-Vis spectroscopy at 273 nm and calculated using a standard curve [7] where $m_{0}$ was the total amount of CP loaded (µg), $m_{1}$ was the amount of free CP (µg), and $m_{CDs}$ was the total amount of CDs (mg).

(2) $$\text{Loading}\text{amount}\;\left(\mu \text{g}\text{CP}\text{mg}{\text{CDs}}^{-1}\right)=\frac{{\text{m}}_{\text{0}}{\text{-m}}_{\text{1}}}{{\text{m}}_{\text{CDs}}}$$

(3) $$\text{Drug}\text{loading}\text{efficiency }\left(\%\right)=\frac{{\text{m}}_{\text{0}}{\text{-m}}_{\text{1}}}{{\text{m}}_{\text{0}}\times 1000}\times \text{100}$$

The binding of CDs with CP was furthermore examined by Matrix-Assisted Laser Desorption Ionization Time-of-Flight/Mass spectroscopy (MALDI-TOF/MS, Autoflex Speed, Bruker, Germany) equipped with a 355 nm Nd:YAG laser operated in the reflection mode. The average cumulative intensity from 1000 laser shots at 2000 Hz was presented with a sampling rate of 2.5 GS s^−1^ for ion detection in the mass range of m/z 0–1000.

### 2.6. Study on pH-sensitive drug release and antimicrobial susceptibility test of CP@CDs

To study the influence of pH on CP released from CP@CDs, 1 mL of CP@CDs was dialyzed using a dialysis membrane (MWCO 1.0 kDa) in 50 mL of 0.1 M PBS buffer solution at pH 5.5 and 7.4 in an incubator shaker at 200 rpm at 37°C. The antibiotic drug release was monitored for the free CP in PBS buffer during 0–48 h measured with UV-Vis spectrophotometer at 273 nm and compared with a standard curve of CP. For the antimicrobial susceptibility test, minimum inhibitory concentration (MIC) and minimal lethal concentration (MLC) of CDs and CP@CDs at different CP loading ratios were measured against the growth and viability of *Escherichia coli* (ATCC 25922), *Pseudomonas aeruginosa* (ATCC 27853) and *Staphylococcus aureus* (ATCC 25923) using a broth dilution method. The procedure followed The Performance Standards for Antimicrobial Susceptibility Testing (30th ed.), Clinical and Laboratory Standard Institute (CLSI) supplement M100.

### 2.7. Cytotoxicity study

The MTT assay was used to investigate the cytotoxicity of CDs samples. For the preparation of the CDs stock solution in a complete culture medium, the stock solution of the CDs was mixed with the culture medium at the ratio of the CDs aqueous solution and 2× concentrated culture medium at 1:1. The final concentration of the CDs stock solutions was: CD280/1 at 1 mg mL^−1^, CP@CDs200 at 100 µg mL^−1^, S-CDs-200C9H at 25 µg mL^−1^, and S-CDs-200C12H at 25 µg mL^−1^.

For MTT assay, 100 µL of the complete culture medium containing L-929 cells (1 × 10^5^ cells mL^−1^ in MEM α), which is mouse fibroblast cell line, was seeded into the well of a 96-well plate and incubated at 37°C in an incubator with 5% CO_2_. After 24 h of cultivation, the culture medium was removed and replaced with the prepared CDs solutions. Different concentrations of the CDs solutions (ranged from 0 to 100%) were tested by diluting the aliquot sample with culture medium. After another 24 h, the culture medium containing CDs was removed, and the cells were rinsed two times with 100 µL of PBS. Then, 100 µL of the MTT reagent was added to the well, and the cells were incubated for 2 h. The MTT reagent was prepared by diluting the stock solution of MTT in PBS (5 mg mL^−1^) 10 times with serum-free DMEM, instead of MEM α, since the MEM α contains ascorbic acid. After the incubation, MTT reagent was removed, and 100 µL of DMSO was added to the well to dissolve the formazan. The 96-well plate was shaken for at least 10 min in an orbital shaker. Finally, the absorbance at 570 nm was recorded with a PerkinElmer EnSpire™ microplate reader (USA). The blank value (using 100 µL of DMSO in the well) was first subtracted from the value of the samples. The cell viability (%) was calculated using Equation (4).

(4) $$Cell\;Viability\left(\%\right)\;=\frac{Absorbance\;of\;the\;sample}{Absorbance\;of\;the\;control}\times 100$$

## 3. Results and discussion

### 3.1. Synthesis and characterization of CDs from a batch hydrothermal method

**Figure 2.**
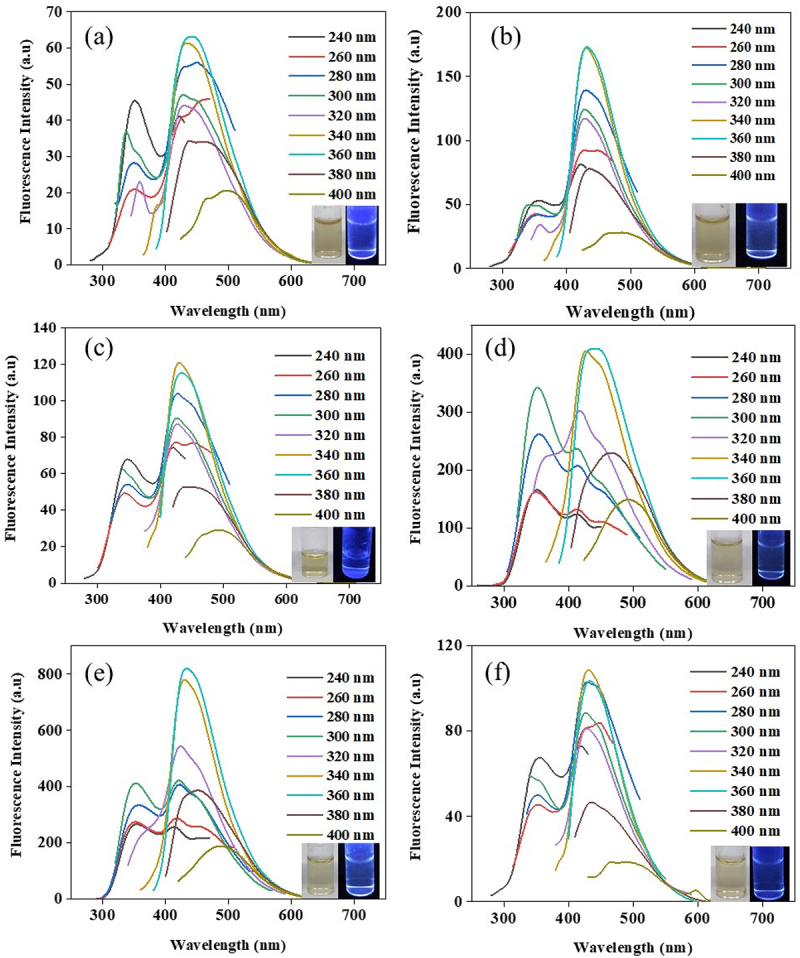
The fluorescence emission spectra of S-CDs synthesized via a batch hydrothermal at vary temperature and time (a) 200C6H (b) 220C6H (c) 200C9H (d) 220C9H (e) 200C12H, and (f) 220C12H at various excitation wavelengths from 240 nm to 400 nm with an interval of 20 nm; inset shows photographs of S-CDs solution under natural light and UV at 365 nm.

**Figure 3.**
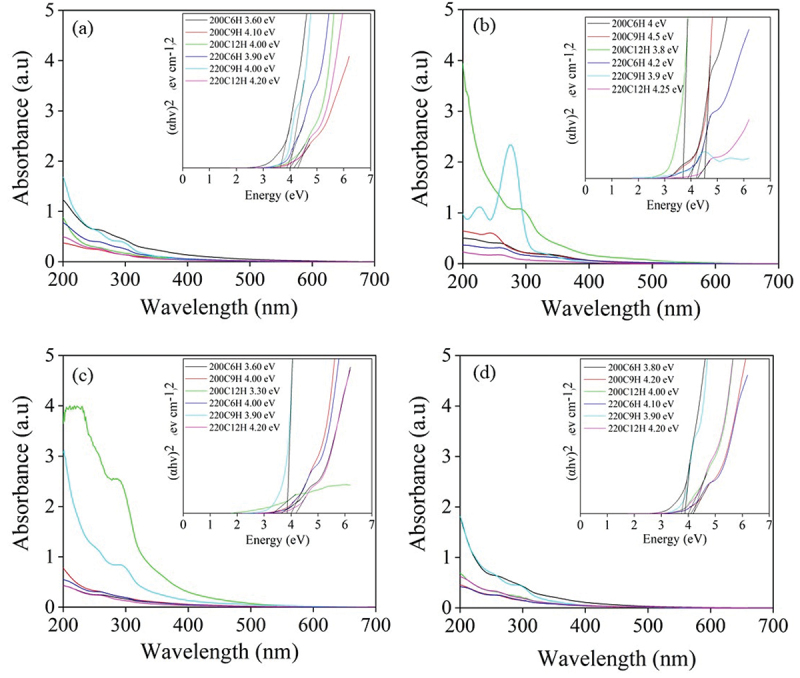
The UV-Visible absorption spectra of (a) X-CDs (b) G-CDs (c) S-CDs and (d) T-CDs with scanned wavelength interval from 200 to 600 nm; inset shows Tauc plots that were used to derive the bandgap.

The CDs were synthesized via a batch hydrothermal process from different sugars in ultrapure water at 200°C or 220°C for 6, 9 and 12 h. The FL properties of xylose-derived CDs (X-CDs), glucose-derived CDs (G-CDs), sucrose-derived CDs (S-CDs) and table sugar-derived CDs (T-CDs) were demonstrated and shown in Figure S1, Figure S2, Figure 2 and Figure S3, respectively. The X-CDs, G-CDs, S-CDs and T-CDs showed the highest emission peak intensity in the range of 430–455 nm with a range of excitation wavelength of 340–360 nm. It was found that CDs synthesized from different sugars exhibited an independent excitation characteristic when varying the excitation from 240 nm to 400 nm. From the result, X-CDs (Figure S1) showed a single emission peak for all excitation wavelengths while G-CDs (Figure S2) showed the FL doublet peak when the reaction time was 12 h at 200°C (Figure S2(e)) and when the temperature was increased to 220°C at 9 h of synthesis (Figure S2(d)). This was possibly due to the formation of sugar molecules to a greater chromophore molecule under hydrothermal reaction at high temperature and pressure which was still in the state of incomplete carbonization. This assumption was accordingly confirmed by UV-Vis absorption peak as shown in Figure 3(b) for the absorption peak at 280–290 nm for G-CDs220C9H and G-CDs200C12H. Figure 2 demonstrates that S-CDs exhibited a doublet peak of emission especially at 220°C for 9 h (Figure 2(d)) and at 200°C for 12 h (Figure 2(e)) similar to T-CDs (Figure S4). The FL doublet peak of sucrose and table sugar corresponded to high UV-Vis absorbance spectra of S-CDs220C9H and S-CD200C12H (Figure 3(c)) as well as those of T-CDs220C9H and T-CD200C12H (Figure 3(d)). The results indicated the presence of n→π* and π–π* interaction between carbon layers of CDs [23] which were analyzed by XPS (Figure 5 and Figures S4-S6) and Raman spectroscopy (Figure 6). In general, the emission wavelength in CDs depends substantially on the presence of diverse functional groups on the surface and the degree of oxidation rather than the particle size [24]. Consequently, the finding was in good accordance with the previous literature.

Figure 3 shows optical absorption spectra that were used to calculate the bandgap by Tauc Plot for CDs derived from all sugars [25]. The Tauc plot was employed for the determination of the CDs’ band gap through the equation, (αhν)^1/n^ = A × (hν–E_g_), where hν represents the energy of the incident photon, A stands for the constant of proportionality, E_g_ signifies the band gap energy, and n takes on the values ½ and 2 for the indirect and direct transition mechanisms, respectively [26]. A direct transition was presumably attributed to electron transition of a single-layer CDs which normally exhibited wider bandgap than an indirect transition owing to either heteroatom or heterojunction of multi-layer CDs [27]. The comparison of the direct and the indirect transition bandgap of S-CDs220C9H was demonstrated in Figure S7 and the result agreed with previous works [26,27]. In this context, the direct transition (*n* = 1/2) was selected to explain the bandgap energy of synthesized CDs due to the fitness of photon absorption and transmittance. Therefore, the plots of (αhν)^2^ as a function of photon energy (E_g_), originating from the transmittance spectra, as shown in the insets of Figure 3 were subjected to fitting using the Tauc model and thus the bandgap energy was read [28]. It was found that the photonic band gap of the synthesized CDs from all sugars at different synthesis conditions was in the similar range of 4.0 to 4.2 eV. Interestingly, narrower band gap less than 4.0 eV (3.6 eV to 4.0 eV) was found in X-CDs, G-CDs, S-CDs and T-CDs synthesized from 200C6H, 200C12H and 220C9H. Detailed structural and optical characterizations revealed that the PL emission was tunable according to the surface state mechanism as well as heteroatom in carbon structure including oxygen, nitrogen, sulfur, etc [20,29]. In addition, the results of XPS data revealed that the oxygen-containing functional groups in CDs, namely, C-OH, C-OOH, C=O, and C=O=C steadily diminished with the red shift of the emission wavelength. This indicated the lessening recombination opportunity of electrons and holes, which was presumably the key reason for the narrowed band gap [30]. Moreover, it was reported that broad band width was accomplished once the CDs were oxidized [31], further evidence is necessary to prove this assumption.

**Figure 4.**
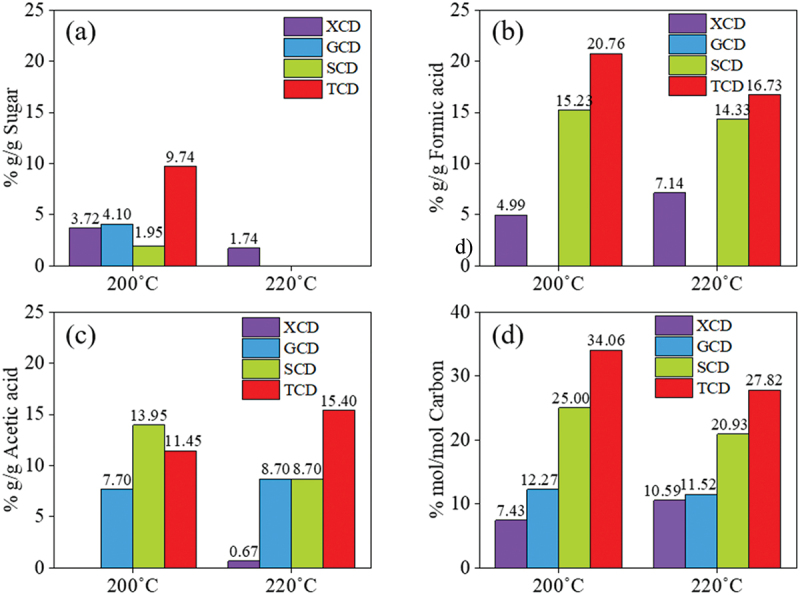
Composition of by-products in aqueous phase during CDs synthesis from different sugars via a batch hydrothermal synthesis reaction at 200°C, 9 h and 220°C, 9 h; (a) residual sugars, (b) formic acid, (c) acetic acid and (d) %carbon recovery in by-products.

Figure 4 shows the HPLC analysis of by-products in the liquid phase collected spontaneously after hydrothermal reaction of all sugars at 200 and 220°C for 9 h. It was found that the residual sugar, *i.e.* table sugar, glucose, xylose and sucrose in a respective degree, was still remained in the CDs solution synthesized at 200°C for 9 h (Figure 4(a)). However, when the temperature was increased to 220°C, sugar in the system was apparently further reacted and converted to CDs and other by-products, and thus no sugar concentration was found in the CDs solution (Figure 4(a)). Moreover, both conditions of CDs synthesis (200°C 9 h and 220°C 9 h) produced substantial amount of formic acid and acetic acid as demonstrated in Figure 4(b,c), respectively. This was possibly because of sugar dehydration to by-products. It was revealed that xylose was transformed to furfural, after which it was converted to furfuryl alcohol and levulinic acid, respectively [32]. In case of sucrose and table sugar, hydrolysis of disaccharides could yield glucose and fructose. It has been reported that glucose and fructose are possibly isomerized and dehydrated during hydrothermal reaction in the presence of acids; therefore, they were further converted to 5-hydroxymethyl furfural (HMF) and subsequently levulinic acid, acetic acid and side-chain formic acid [33], in which the present work was detected as a major by-product by HPLC [34]. Besides, levulinic acid is possibly reacted with molecular oxygen to yield acetic acid in water at high temperature [34,35]. Apart from HPLC result, LC/MS/MS Orbitrap analysis revealed that sucrose can be converted to 5-Hydroxymethyl-2-furaldehyde (HMF), the main product. HMF was apparently converted to furfuryl alcohol or furfuranol (Tables S1 and S2). After that the furfuryl alcohol seemingly reacts to form acetic acid and appeared in the form of 2-[3-methyl-2-(methylimino)-4-oxo-1,3-thiazolan-5-yl]acetic acid as demonstrated. Apart from HMF, the LC/MS/MS results exhibited the presence of furan derivatives in the reaction solution after S-CDs synthesis such as furan-2,5-dicarbaldehyde, 4-hydroxy-5-methyl-3-furanone, 4-Oxo-4,5,6,7-tetrahydro-1-benzofuran-3-carboxylic acid, furan, 5-phenoxy-2-furoic acid and furfuranol (Table S1 and S2). In case of carbon content as shown in Figure 4(d), an increased synthesis temperature from 200 to 220°C could decrease carbon recovery in by-products, *i.e.* residual sugars, formic acid and acetic acid. This was presumably because carbon dioxide can be generated during the reaction and evaporated into incondensable gaseous product [33]. The highest loss of carbon into acids and remaining sugars was found in table sugar, sucrose, glucose and xylose in a respective degree.

**Figure 5.**
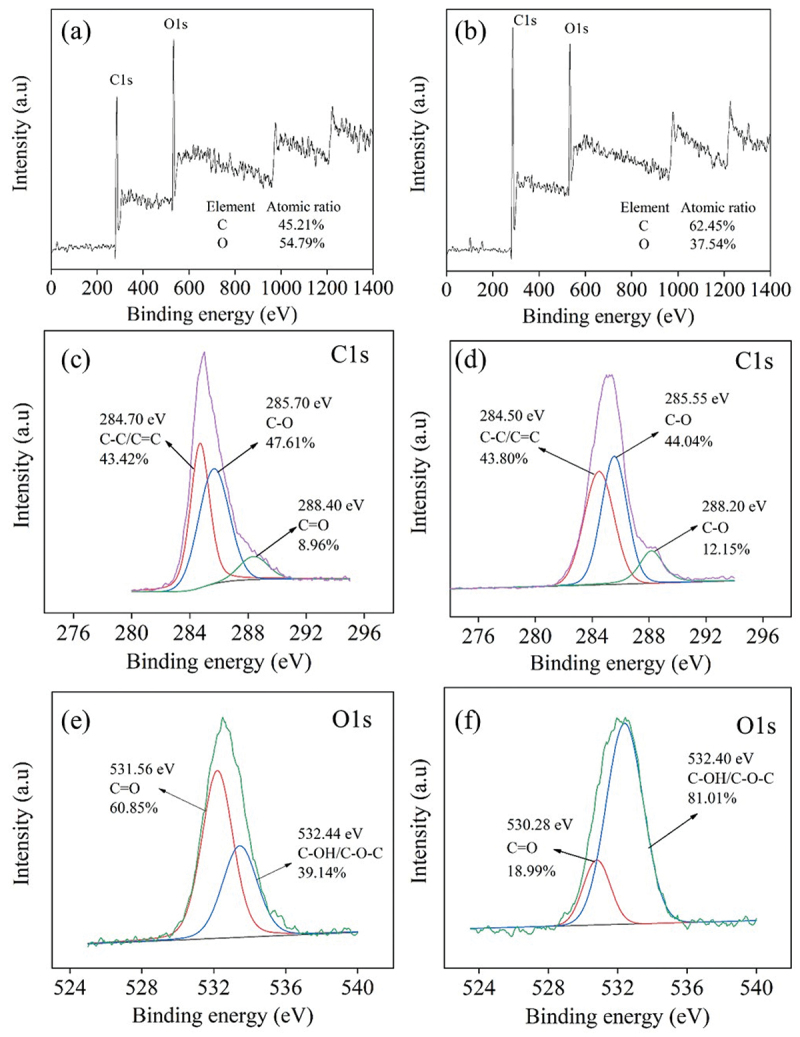
The XPS analysis of S-CDs (a-b) survey scan 200°C and 220°C, (c-d) C1s 200°C, and 220°C, (e-f) O1s 200°C and 220°C when the synthesis time was 9 h.

The XPS analysis of the surface state of S-CDs (Figure 5) showed that the full-scanned XPS spectrum of S-CDs was mainly composed of C and O elements at binding energy 285 eV and 532 eV, respectively, for 200°C 9 h synthesis condition (Figure 5(a)), and the binding energy of 286 eV and 533 eV, respectively for 220°C 9 h synthesis condition (Figure 5(b)). From Figure 5(c), the high resolution of C 1 s peak for S-CDs synthesized at 200°C for 9 h showed three deconvoluted peaks at 284.7 eV, 285.7 eV and 288.4 eV assigned to C-C/C=C, C-O and C=O [33], respectively. Similarly, the energy class of C 1s of S-CDs synthesized at 220°C for 9 h observed in Figure 5(d) consists of three deconvoluted peaks at 284.5 eV, 285.6 eV and 288.2 eV which were assigned for C-C/C=C, C-O and C=O [33], respectively. Finally, Figure 5(e) demonstrated the surface state of O 1s for S-CDs 200C9H revealing two peaks at 530.28 eV and 532.4 eV which were assigned for C=O and C-OH/C-O-C [33], respectively. Likewise, O 1 s deconvoluted peaks of S-CDs 220C9H synthesized at 220°C for 9 h (Figure 5f) were observed at 530.3 eV and 532.4 eV assigned to C=O and C-OH/C-O-C, respectively. From atomic content analysis based on XPS results, the percentage of carbon atoms relative to other constituents was substantially increased from 45.2% to 62.5% (Figure 5(a,b)) when the synthesized temperature increased from 200°C to 220°C at the same synthesis time of 9 h indicating greater extent of condensation and subsequent carbonization reaction from which oxygen and hydrogen were eliminated from core carbon structure of CDs.

Similar XPS results of the surface state of CDs were obtained from X-CDs, G-CDs and T-CDs as shown in Figure S4, S5 and S6, respectively. From atomic content analysis, the percentage of carbon atoms relative to other constituents of X-CDs and T-CDs was increased from 52.7% to 58.3% and from 47.7% to 58.8%, respectively, when the synthesized temperature increased from 200°C to 220°C at the same synthesis time of 9 h. This indicated a greater extent of carbonization reaction from which oxygen and hydrogen were eliminated from carbon structure of CDs. In contrast, a decrease of atomic carbon content from 54% to 47.9% was observed for G-CDs when the synthesized temperature increased from 200°C to 220°C at 9 h of synthesis time. This was mainly due to a greater oxidation state of G-CDs compared with X-CDs, S-CDs and T-CDs. Therefore, it was presumably referred that higher extent of functional groups including carboxyl groups, carbonyl groups and hydroxyl groups may be formed and adhered onto the surface of the G-CD [35].

**Figure 6.**
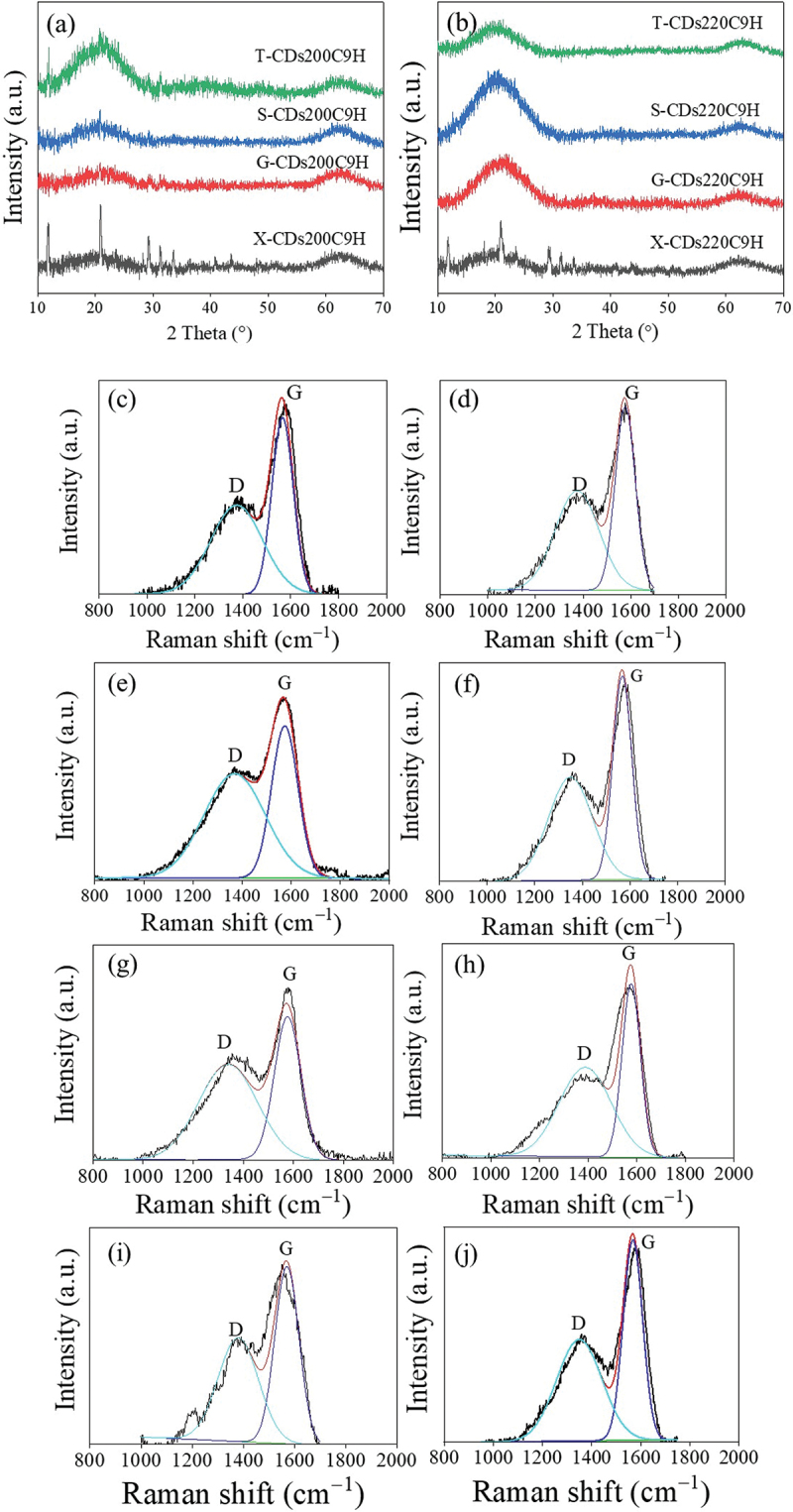
XRD pattern of CDs synthesized at (a) CDs at 200°C (b) CDs at 220°C for 9 h, and Raman spectral profile of (c) X-CDs200C9H, (d) X-CDs220C9H, (e) G-CDs200C9H, (f) G-CDs220C9H, (g) S-CDs200C9H, (h) S-CDs220C9H, (i) T002DCDs200C9H and (j) T-CDs220C9H of batch hydrothermal synthesis of CDs.

Crystal morphology of all synthesized CDs from different sugars at varied synthesis temperatures was analyzed using XRD and Raman spectroscopy as shown in Figure 6. For XRD diffractograms of CDs synthesized at 200°C for 9 h (Figure 6(a)), an obvious peak assigned to 002 graphitic crystal plane was detected for all CDs samples at 2θ = 20.8–20.9° [36,37]. It has been found from XRD intensity that when the synthesized temperature increased from 200°C (Figure 6(a)) to 220°C (Figure 6(b)), X-CDs, G-CDs and S-CDs have a higher crystallinity, and especially S-CDs exhibited considerably higher crystallinity than G-CDs and X-CDs. Nevertheless, the crystallinity of T-CDs synthesized at 220°C for 9 h was seemingly lower than T-CDs synthesized at 200°C for 9 h. The result agreed with the ratio of integrated peak area of Raman spectra between G band and D bands of T-CDs as shown in Table 1.

**Table 1.** Integrative peak area of D and G band of carbon structure of synthesized CDs via a batch hydrothermal method at different temperatures for 9 h.

CDs sample	I_D_ (cm^−1^)	I_G_ (cm^−1^)	I_D_/I_G_
X-CDs 200C9H	461884.80	370309.10	1.24
X-CDs 220C9H	751365.20	629900.40	1.19
G-CDs 200C9H	902106.40	566918.00	1.59
G-CDs 220C9H	874772.50	741382.80	1.17
S-CDs 200C9H	327889.80	228027.00	1.43
S-CDs 220C9H	1022648.00	902119.30	1.13
T-CDs 200C9H	426663.60	436245.00	0.97
T-CDs 220C9H	1199994.00	720127.80	1.66

From the results, the Raman spectra were in good agreement with XRD patterns of all CDs that represent the graphitic or crystalline carbon through G band at 1590 cm^−1^ and the amorphous carbon corresponding to D band at 1390 cm^−1^. As demonstrated in Figures 6(c–j), the Raman peaks of D and G bands of synthesized CDs appeared in all synthesis conditions with different intensities, and the calculated I_D_/I_G_ values from integrative peak areas from D band and G band are shown in Table 1. The presence of G band confirms the existence of a crystalline core with sp^2^ hybridization, while the D band indicates the presence of defects and disordered carbon typically associated with amorphous carbon. When increasing synthesis temperature from 200°C to 220°C for X-CDs, G-CDs and S-CDs, lower I_D_/I_G_ values were obtained which designated the more crystalline or graphitic plane arrangement of core carbon (G band) compared with amorphous structure (D band). However, the graphitic plane arrangement of T-CDs decreased when the synthesis temperature increased demonstrating more amorphous structure which was possibly caused by high degree of oxidation or O-doped CDs. Evidently, O-CDs were found to be the greatest disorder compared with N-CDs and S-CDs from the previous research. This agreed with the absence of well-noticeable UV-Vis absorption peaks of T-CDs (Figure 3(d)) since these are associated with the crystalline, sp^2^ hybridized carbon found in the CDs core [38]. From the molecular modeling study, the UV-Vis absorption study of O-CDs, using a straightforward multilayer model comprising oxygen-functionalized coronene and pyrene building blocks, revealed that the aromatic carbon rings form a conjugated sp^2^ system [23]. This system exhibits overlapping when multiple building blocks are stacked together. The oxygen-doped carbon structure was also confirmed by a high-energy UV absorption band of π→π* feature at 260 nm. A lower energy band near 300 nm absorption attributed to the interlayer π→π* charge transfer on its high energy side and to n→π* shifts on its lower energy side. The sp^2^-hybridized carbon core caused π→π* transitions while n→π* are due to non-binding electron orbitals, introduced by the oxygen functionalization at the CD edge [38]. Therefore, the analysis of Raman spectra and XRD patterns is consistent with UV-Vis absorbance as the optimal synthesis temperature and time. X-CDs, G -CDs and S-CDs exhibited a higher crystallinity owing to their enhanced graphic carbon structure relative to disordered or amorphous carbon structure. In case of T-CDs, high temperature at 220°C for 9 h may cause strong oxidation and the crystalline carbon structure could be damaged.

**Figure 7.**
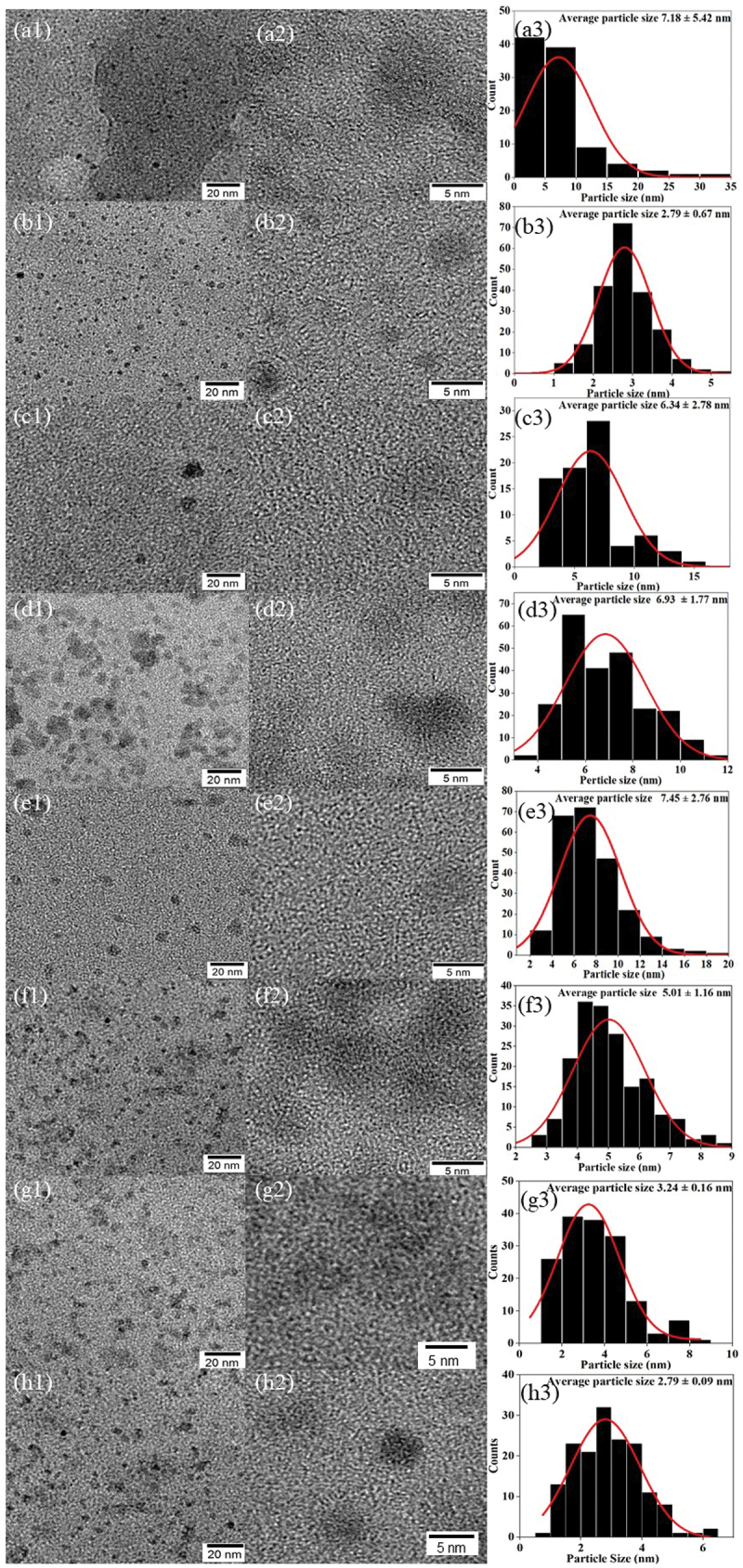
TEM images with particle size distribution of CDs (a) X-CDs220C6H, (b) X-CDs220C12H, (c) G-CDs220C6H, (d) G-CDs220C12H, (e) S-CDs220C6H, (f) S-CDs220C12H, (g) T-CDs220C6H and (h) T-CDs220C12H.

In Figure 7, TEM images and the corresponding size distribution of X-CDs, G-CDs, S-CDs and T-CDs show that all CDs samples are homogeneous and consistent with the average particle size between 2.8 and 7.5 nm with spherical geometry. When the synthesis temperature was 220°C and the reaction time was increased from 6 h to 12 h, the average size of X-CDs, S-CDs and T-CDs was found to be decreased from 7.2 ± 5.4 nm to 2.8 ± 0.7 nm (Figure 7(a,b)), from 7.5 ± 2.8 nm to 5.0 ± 1.1 nm (Figure 7(e,f)), and from 3.2 ± 0.2 nm to 2.8 ± 0.1 nm (Figure 7(g–h)), respectively. The average size of G-CDs was not changed with an increased reaction time, and the size was in a range of 6.3 ± 2.8 nm and 6.9 ± 1.8 nm for the G-CDs220C6H and G-CDs220C12H (Figure 7(c,d)), respectively.

**Figure 8.**
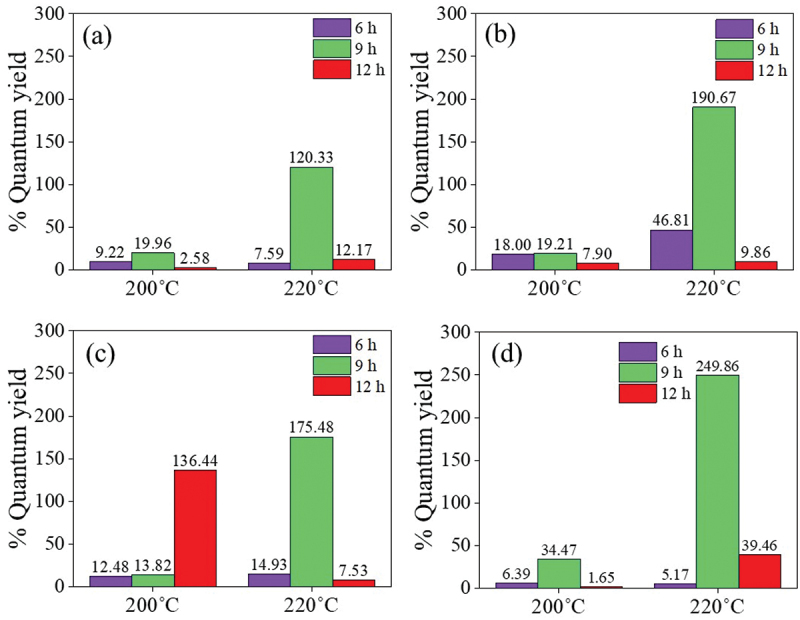
The %QY of (a) X-CDs, (b) G-CDs, (c) S-CDs and (d) T-CDs via a batch hydrothermal method when varying temperatures and times for CDs synthesis at 200°C and 220°C for 6, 9 and 12h.

**Figure 9.**
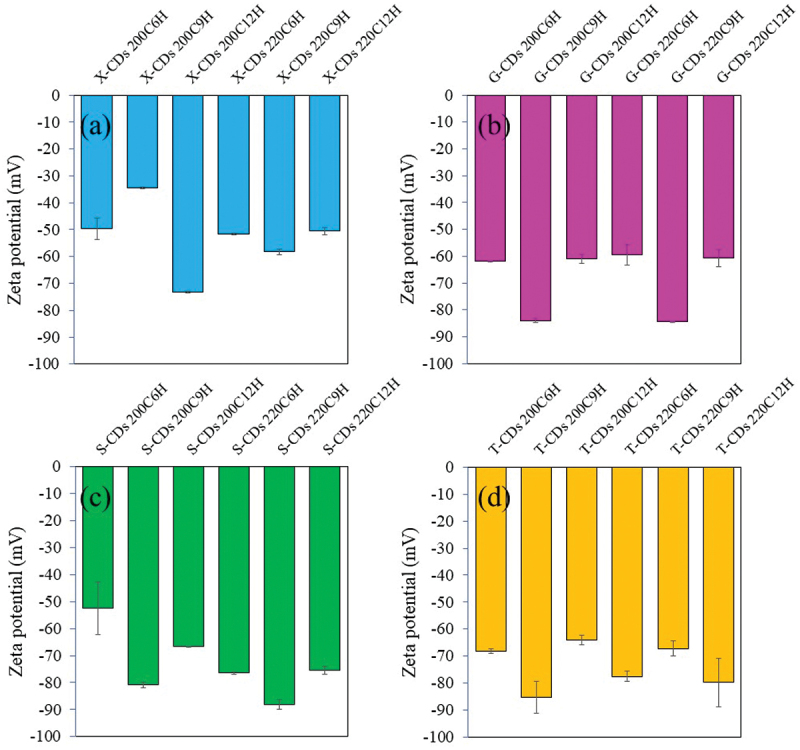
The zeta potential of (a) X-CDs, (b) G-CDs, (c) S-CDs and (d) T-CDs via a batch hydrothermal method at varying temperatures and times for CDs synthesis.

From the FL and UV-vis absorbance analysis of CDs synthesized from different sugars via a batch hydrothermal method, the %QY can be calculated relative to quinine sulfate as the reference (%QY = 54). As shown in Figure 8, the synthesis condition of 220°C for 9 h was the optimal condition for CDs synthesis from all investigated sugars providing the highest %QY compared with other synthesis conditions. The %QYs of X-CDs, G-CDs, S-CDs and T-CDs were 120.3%, 190.7%, 175.5% and 240.9%, respectively. The high QY over 100% was due to the greater emission intensity of CDs compared with quinine sulfate when the concentration of both CDs and the reference was the same. The result of zeta potential in Figure 9 shows the highly negative charge of all CDs representing high stability of nanoparticles suspended in the aqueous phase due to the electrostatic repulsion among individual particle. The sufficient repulsive force to attain better physical colloidal stability was reported through a zeta potential value over −30 mV or +30 mV [39]. The high value of negative charge of zeta potential for all CDs (Figure 9) additionally demonstrated an effective binding with cations such as heavy metal ions [20,40] and ability to conjugate with positively charged drugs such as CP [7] for biomedical applications.

In addition to the study of CDs properties, the %mass yield of a product is another factor that should be considered. The %mass yield of CDs produced via a batch hydrothermal method calculated based on the initial dry weight of sugar precursor is shown in Figure S8. The highest %mass yield of CDs was achieved from S-CDs. At the synthesis condition of 200°C 12 h and 220°C 9 h, the mass yields of S-CDs200C12H and S-CDs220C9H were 2.62% and 2.56%, respectively, which were obviously greater than that of CDs synthesized from other sugars from all synthesis conditions. Since S-CDs from sucrose exhibited most excellent efficiency in all aspects, namely, high QY, highest mass yield, supreme crystallinity through the highest peak area of I_G_ from Raman spectra, greatest stability due to highest zeta potential and utmost carbon content of CDs from XPS analysis; therefore, sucrose was chosen as a precursor for the study in a flow reactor system for CDs synthesis via a continuous hydrothermal process.

### 3.2. Synthesis and characterization of S-CDs from a continuous flow reactor system

**Figure 10.**
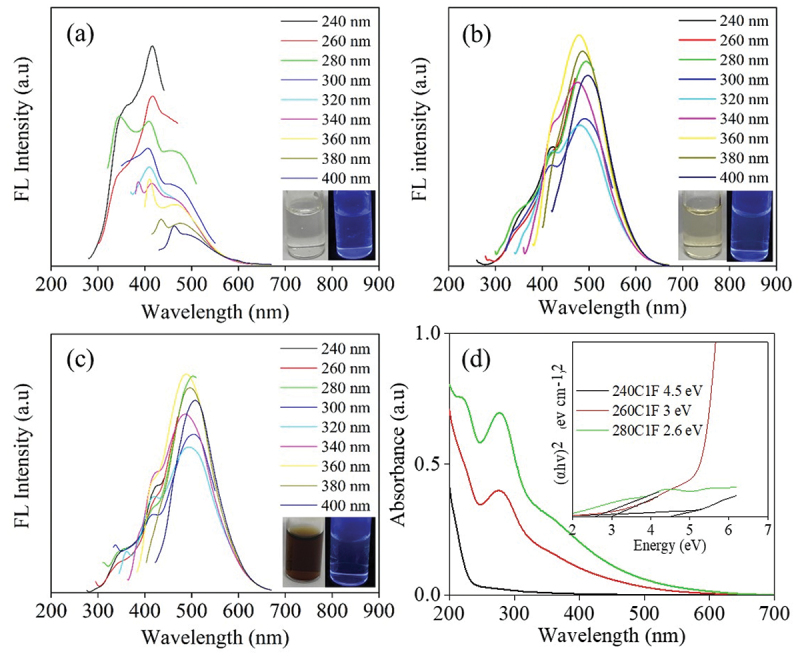
The fluorescence emission spectra of S-CDs synthesized via a continuous hydrothermal at a flow rate of 1 mL min^−1^ and varied temperatures: (a) 240°C (b) 260°C (c) 280°C at various excitation wavelengths from 240 nm to 400 nm with an interval of 20 nm, and (d) the UV-visible absorption spectra of the S-CDs at flow rate 1 mL min^−1^ with a scanned wavelength from 200 to 600 nm; inset show optical bandgap from Tauc plot.

The S-CDs was synthesized in a flow reactor via a continuous hydrothermal process from sucrose solution (5%w/v) at different residence time and the varying flow rate (1, 5, and 10 mL min^−1^) which was corresponded to the residence time of 4.4, 0.9 and 0.4 min, respectively. The synthesis temperature was varied in a range from 240 to 350°C. Figures 10, S9 and S10 show the FL and optical absorption spectra of S-CDs synthesized at various temperatures from 260 to 280°C in a continuous system at the flow rate of 1 mL min^−1^, 5 mL min^−1^ and 10 mL min^−1^, respectively. As demonstrated in Figure 10(a), the S-CDs synthesized at a flow rate of 1 mL min^−1^ exhibited the strongest FL emission intensity at 410 nm under 240 nm excitation when the synthesis temperature was 260°C. At the synthesis temperature of 240°C (Figure 10(a)), FL was weak and the peaks varied with excitation wavelength. This was referred to either incomplete carbonization of CDs or the presence of oxygen containing functional groups. However, when the synthesis temperature increased to 260 and 280°C, the stronger FL emission of S-CDs was achieved. FL emission at ~ 480 and ~490 nm was observed under 360 nm excitation of S-CDs from the reaction temperature of 260 and 280°C at 1 mL min^−1^ flow rate as demonstrated in Figures 10(b,c), respectively. This implied that the red-shift in the FL emission was obviously detected from the maximum emission wavelength of 410 nm (Figure 10(a)) to ~480 nm (Figure 10(b)) and ~490 nm (Figure 10(c)), when increasing the reaction temperature from 240 to 260 and 280°C, respectively.

The UV-Vis absorption, as shown in Figure 10(d), presented two absorption peaks of S-CDs, when increasing the reaction temperature at a flow rate of 1 mL min^−1^. The UV-vis absorption band at 282–283 nm was related to the $n-\pi^{*}$ transition for C=O moieties that are abundantly located in the CDs surface [13] while the absorbance at 225 nm is assigned to aromatic sp^2^ $\pi -\pi^{*}$ transition within the graphitic core (C=C and C-C bonds) [13]. In addition, the Tauc plots indicated [36] that the bandgap energy of the synthesized S-CDs at a flow rate of 1 mL min^−1^ decreased from 3.0 eV to 2.6 eV when the reaction temperature increased from 260°C to 280°C. The optical bandgap additionally specifies either an increase of CDs size when synthesis temperature increased or a red shift of FL from ~410 nm and 260°C (Figure 10(b)) to ~490 nm and 280°C synthesis temperature (Figure 10(c)) when the similar excitation wavelength of 340–380 nm was applied. From this circumstance, the oxidation of carbon structure to form oxygen-containing functional groups on the surface, so-called surface defects, apparently played an important role on trapping and emitting light at different wavelengths. It was reported that the quantity of surface functional groups existing on CDs was influenced by the degree of oxidation, and an increased surface oxidation can result in a shift toward longer wavelengths in the emitted light [41,42]. It was furthermore reported that the larger π-conjugated domains can cause the band gap smaller, which lead to the red shift in the emission peak [43].

**Figure 11.**
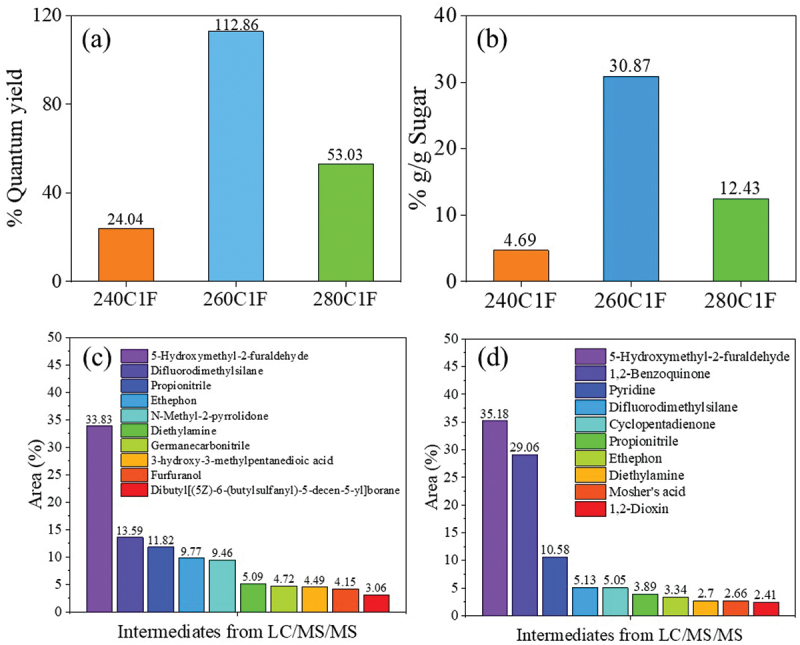
(a) the %QY of S-CDs, (b) % carbon content in by-products during CDs synthesis at 1 mL min^−1^ and varying temperature in a range of 240–280°C, and chemical composition of by-products from LC/MS/MS analysis from S-CDs synthesis from the continuous hydrothermal synthesis reaction at 1 mL min^−1^ at (c) 260°C, and (d) 280°C via a continuous hydrothermal method at a flow rate of 1 mL min^−1^.

When the flow rate was increased from 1 mL min^−1^ to 5 mL min^−1^ and 10 mL min^−1^, the residence time was substantially decreased and therefore S-CDs was not completely formed. The reason was due to an insufficient time of reaction for CDs to be shaped by a combination of polymerization, condensation and carbonization [16,44]. This was confirmed by the FL properties of synthesized CDs (Figures S9(a)-S9(c) and Figures S10(a)-S10(c)). The %QY of synthesized S-CDs in a continuous system at a flow rate of 1 mL min^−1^ at different temperatures between 240 and 280°C was calculated based on the FL and UV-vis absorbance values. In Figure 11(a), the highest %QY (%QY = 112.8%) was obtained from S-CDs synthesized at a temperature of 260°C at a flow rate of 1 mL min^−1^ which was twice as much as the %QY of S-CDs synthesized at 280°C (%QY = 53%) and 240°C (%QY = 24%). Sucrose residue was still observed in the CDs solution (Figure 11(b)), and HPLC chromatograms in Figure S11(a – c) indicate the presence of unknown intermediates and polymeric products (retention time between 5 and 10 min) when increasing synthesis temperature from 240 to 260 and 280°C. The carbon recovery calculated from sugar and organic acids detected in liquid aliquot as well as the carbon content in S-CDs is shown in Figure S11(d). The result revealed that carbon recovery from synthesis at 280°C (36.13%mol/mol) was greater than that at 260°C (31.48%mol/mol) and 240°C (4.69%mol/mol). This was possibly owing to an incomplete carbonization of carbon source to CDs at lower synthesis temperature.

To elucidate the intermediates formed during S-CDs synthesis via a continuous hydrothermal method at the flow rate of 1 mL min^−1^ at 260 and 280°C, the LC/MS/MS Orbitrap technique was applied. As shown in Figure 11(c–d), the top-tenth substances mainly detected as by-products during S-CDs synthesis at 260 and 280°C were qualitatively observed, respectively. It was observed that the compounds with molecular mass between m/z 98.9 and 126.7 were considerably formed at 260 and 280°C, which could be identified as 5-hydroxymethyl-2-furaldehyde (HMF) [33]. From both the reaction temperatures, the highest concentration of HMF detected was due to glucose and fructose dehydration reaction in the presence of molecular oxygen in acidic condition. HMF which was the main product detected by LC/MS/MS was possibly converted to furfuryl alcohol or furfuranol (Table S1 and S2). After that the furfuryl alcohol apparently reacts to form acetic acid and appeared in the form of 2-[3-methyl-2-(methylimino)-4-oxo-1,3-thiazolan-5-yl]acetic acid. In summary, a variety of furan derivatives from sucrose was observed through LC/MS/MS Orbitrap at different degrees influenced by synthesis temperature and time as demonstrated in Table S1 and S2, for instance, 5-Hydroxymethyl-2-furaldehyde, furan-2,5-dicarbaldehyde, 4-Hydroxy-5-Methyl-3-Furanone, 4-Oxo-4,5,6,7-tetrahydro-1-benzofuran-3-carboxylic acid, furan, 5-Phenoxy-2-furoic acid and furfuranol. As shown in Figure 11(c–d), difluorodimethylsilane, propionitrile and ethephon were also found in substantial amount from both reaction temperatures. This was possibly due to the methanol formation which was generated by formic acid [45] that can react with silicon [46]. Meanwhile, acetic acid reversibly reacts with either amide group [47], or ethylene [48], and after that ethylene is converted to ethephon [49], accordingly.

**Figure 12.**
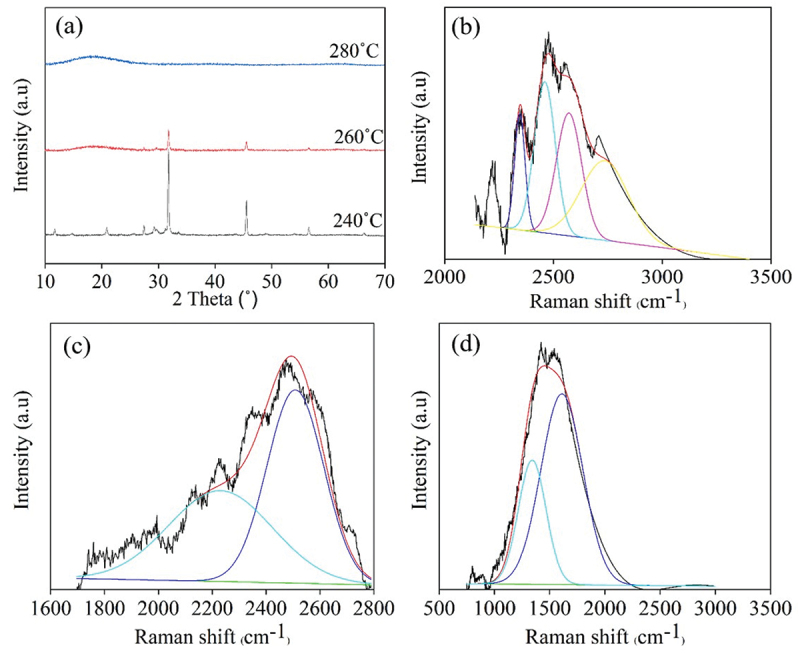
(a) XRD patterns, and Raman spectral profile of S-CDs synthesized via a continuous hydrothermal synthesis by 1 mL min^−1^ at (b) 240°C (c) 260°C and (d) 280°C.

From Figure 11, the least residual sucrose was quantified at 240°C reaction temperature at a flow rate of 1 mL min^−1^ while higher temperature (260°C and 280°C) may cause greater evaporation of water and yielded higher concentration of sucrose residue. Besides, an enhanced dehydration of sucrose to acids, furan derivatives as well as polymeric compounds markedly occurred at 260°C and 280°C. Moreover, at elevated temperature, carbon nanodots are substantially formed by an increased carbonization by deoxygenation of sugar and acids [34]. This statement was confirmed by a transformation of sugar crystals observed by XRD patterns at 2θ equal to 32° from S-CDs synthesized at 240°C to a broad peak of XRD patterns of carbon nanostructure at 2θ equal to 18° from S-CDs synthesized at 260°C and 280°C (Figure 12(a)). For S-CDs synthesized at 260°C and 280°C, XRD peaks of 002 plane found at 2θ equal to 18°Correspond to the graphitic carbon structure of CDs [50]. However, the XRD peak at 2θ equal to 32° was slightly detected for S-CDs synthesized at 260°C which were assigned to residual sucrose [51].

In addition, Raman spectra of sugar peak at around 2400 to 2600 cm^−1^ were shifted to carbon peaks at 1390 and 1590 cm^−1^ for D and G bands of amorphous and crystalline carbon structure of S-CDs, respectively, when increasing reaction temperature from 240°C (Figure 12(b)) and 260°C (Figure 12(c)) to 280°C (Figure 12(d)). The Raman spectra of S-CDs synthesized at 240°C and 260°C demonstrated the Raman peaks at 2470 cm^−1^ (Figure 12(b,c)) assigned to sucrose structure. Therefore, it was referred that the synthesized CDs underwent incomplete carbonization at 240°C and 260°C, and the sucrose precursor was possibly hydrolyzed and dehydrated to generate other intermediates [33]. Raman spectra of S-CDs synthesized at 280°C (Figure 12(d)) exhibited the I_D_/I_G_ ratio of 0.43 which was much lower than those synthesized by batch hydrothermal system (Table 1). This corresponds to the high crystallinity of carbon structure as a result of the high content of graphitic carbon structure found in S-CDs from a continuous process compared with a batch process [50].

The Raman spectra of S-CDs synthesized from sucrose at 260°C, 280°C, 300°C, 325°C and 350°C using 5 mL min^−1^ and 10 mL min^−1^ flow rates exhibited unreacted sucrose in a solid form found at 2900 cm^−1^ [52] as shown in Figure S12 and Figure S13, respectively. The reason was mainly because of an insufficient reaction time (residence time of 0.9 min for 5 mL min^−1^ flow rate, and 0.4 min residence time for 10 mL min^−1^ flow rate). Therefore, incomplete polymerization, condensation and carbonization due to short residence time could occur in the flow reactor system with high flow rate [16].

In Figure S14, TEM images and the corresponding S-CDs size distribution from the continuous hydrothermal process show that two samples were well dispersed with an average particle size of ~ 11.9 ± 0.8 nm and ~ 6.5 ± 0.6 nm with nearly spherical geometry when the synthesis temperature was 260°C and 280°C, respectively [50]. The carbon lattice spacing was not obviously seen at these reaction temperatures. The result of the zeta potential (Figure S15(a)) showed that the negative zeta potential of the S-CDs synthesized at a flow rate of 1 mL min^−1^ was decreased when the reaction temperature was increased. From the zeta potential values, all S-CDs synthesized at a flow rate of 1 mL min^−1^ showed high stability of nanoparticles in the aqueous phase. Since it has been reported that CP molecule becomes positively charged because of the protonation of the amine group [53], consequently negatively charged S-CDs exhibited a possibility on effectively conjugating with CP [7] for antimicrobial drug delivery application. The %mass yields of S-CDs synthesized via a continuous hydrothermal process at a flow rate of 1 mL min^−1^ at various temperature from 240 to 280°C are shown in Figure S15(b). The % S-CDs mass yields of 3.4, 5.0 and 13.37%, were obtained when the reaction temperature was 240, 260 and 280°C, respectively. The result was consistent to the carbon recovery as demonstrated in Figure S11(d).

### 3.3. Study on CP@CDs conjugation, the controlled release of CP at different pHs and antimicrobial susceptibility of CP@CDs

**Figure 13.**
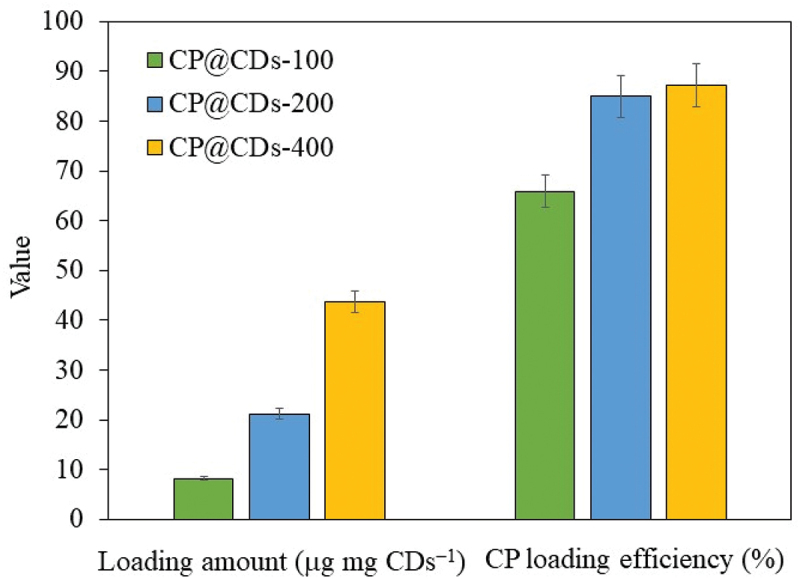
The loading amount and %CP loading efficiency of CP@CDs-100, CP@CDs-200 and CP@CDs at 24 h, 25°C in a shaking incubator at 200 rpm.

The S-CDs synthesized from sucrose via a continuous flow reactor system at 280°C and a flow rate of 1 mL min^−1^ was applied in a drug-delivery system by conjugation with CPCP, which is an antimicrobial drug capable of inhibiting mainly gram-negative bacteria such as *Proteus vulgaris, Escherichia coli, Klebsiella pneumoniae* [54], *Pseudomonas aeruginosa*, and some gram-positive bacteria such as *Staphylococcus aureus* [55]. The loading amount of CP and %CP loading efficiency at different loading ratios of S-CDs to CP is demonstrated in Figure 13. The concentration of CP for the conjugation with CDs was 100, 200 and 400 mg mL^−1^, and named as CP@CDS-100, CP@CDS-200 and CP@CDS-400, respectively. Figure 13 shows that the loading amount of CP@CDS-100, CP@CDS-200 and CP@CDS-400 increased accordingly with an increase of CP concentration added for the conjugation with CDs. The %CP loading efficiency for CP@CDS-100, CP@CDS-200 and CP@CDS-400 was 65.9%, 84.9% and 87.2%, respectively, for 24 h loading duration at 25°C in a shaking incubator at 200 rpm. The results showed that the S-CDs synthesized via a continuous flow reactor system was an effective nanocarrier as a CP delivery agent when compared with other carrier system, as shown in Table 2. An additional characterization of CP@CDs-200 with MALDI-TOF/MS technique to confirm the conjugation between CP and S-CDs is shown in Figure S16. S-CDs synthesized from sucrose by a continuous flow reactor system showed the mass to charge ratio (m/z) in the range of 0–100 and 380–480. This indicated that the S-CDs were composed of C, H and O atoms in the m/z range of 100 to 380, especially the pronounced peak at m/z 225 was observed. While the S-CDs conjugated with CP (CP@CDs-200) showed the mass-charge ratio (m/z) especially at 215, 248, 322 and 354, which revealed that CP was loaded onto the surface of S-CDs [56].

**Table 2.** A comparison of the %CP loading efficiency of the present study and other carrier systems.

Carrier	CP loading efficiency (%)	Ref.
κ-carrageenan-cross-linked Chitosan/hydroxyapatite hydrogel nanocomposites	79.5	[1]
sodium alginate/starch blends	67.0	[2]
N@CDs	6.0	[3]
CP@CDs-100	65.9	This work
CP@CDs-200	84.9	This work
CP@CDs-400	87.2	This work

**Figure 14.**
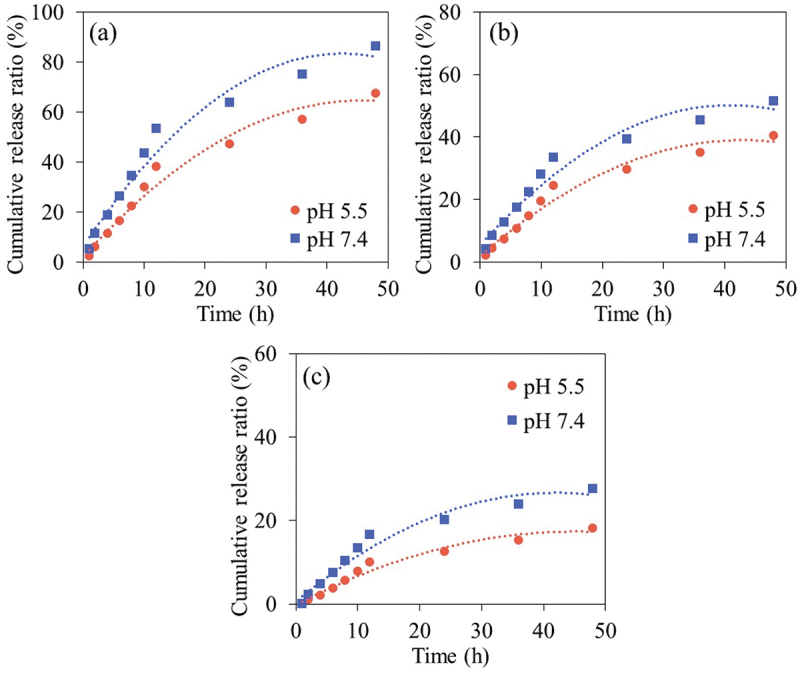
The cumulative CP release profile from (a) CP@CDs-100, (b) CP@CDs-200, and (c) CP@CDs-400 conjugates at different pH from 0 to 48 h at 37°C in an incubator shaker at 200 rpm.

The study of %CP release was conducted at pH 5.5 and 7.4 using phosphate buffer solution (PBS) for CP@CDs-100, CP@CDs-200 and CP@CDs-400. Figures 14(a–c) shows that the cumulative release profile of CP from all CP@CDs conjugates exhibited an increasing release trend with a longer time. An increase of pH from 5.5 to 7.4 was found to enhance the CP release capability for all samples (CP@CDs-100, CP@CDs-200 and CP@CDs-400) which was in good agreement with a previous work [57]. The cumulative CP release profile of CP@CDs can be divided into two periods. In the initial period (0–12 h), the CP@CDs released rapidly at pH 5.5 and 7.4 for all formulations before the release capacity of CP@CDs reached the onset steady state in the second period (12–48 h) [7]. The CP@CDs-100 unveiled a higher %CP release at all pH and it was followed by CP@CDs-200 and CP@CDs-400, respectively. The CDs synthesized via a continuous flow reactor system had a negative charge but a relatively low zeta potential value. When the CDs conjugated with CP under acidic pH (pH 5.5) to neutral pH 7, the negative charges on the surface of the CDs were presumably altered to positive charges. As a result, at such pH conditions, the binding strength of CP on the CDs was reduced, leading to a higher release of the drug. Accordingly, the CDs conjugated with CP at low concentration formulation (e.g. CP@CDs-100) are released faster than the CDs conjugated with CP at high concentration formulation (e.g. CP@CDs-400) [58]. In addition, it was found that the release of drug was greater at pH 7.4 compared to pH 5.5 of all three samples. The reason is possible because the carboxyl functional groups of CP in the form of -COOH at pH 5.5, strongly bind with CDs, and thus inhibit the drug release at pH 5.5 or in the acidic pH range [59]. For further biomedical applications, antibiotic activity and cytotoxicity test were performed to confirm the effectiveness of CP@CDs conjugates for the drug-delivery implementation in anti-inflammatory, antibiotic and nano-theranostic applications.

To investigate antibacterial effect, the antimicrobial susceptibility test was performed and reported as minimal inhibitory concentration (MIC) that is defined as *in vitro* levels of susceptibility of specific bacterial strains to antimicrobial agent, and minimal lethal concentration (MLC) that is defined as the maximum dilution of the product that kill the test organism. As shown in Table 3, MIC and MLC of CDs and CP@CDs at different loading ratios were reported against the growth of *Staphylococcus aureus* ATCC 25923, *Pseudomonas aeruginosa* ATCC 27853, and *Escherichia coli* ATCC 25922. These findings suggested that least inhibitory effect (MIC) was observed for CDs synthesized from sucrose toward all microorganisms tested, and CDs did not kill the bacteria at even high concentration at 250 mg mL^−1^. At high CP loading ratio, CP@CDs-400 showed ability to suppress the growth of *E. coli* > *P. aeruginosa > S. aureus* indicating the effect of CP on gram-negative strains due to high release rate of CP at the initial state, however at lower loading ratio of CP@CDs-100 and CP@CDs-200 that released lower rate of CP at the initial state, the inhibitory effect was found for *E. coli > S. aureus* > *P. aeruginosa*. In terms of MLC, no effect of all CP@CDs on inactivation of *S. aureus* while little effect of CP@CDs against *E. coli > P. aeruginosa* was observed. The results in this study unveiled that antimicrobial resistance of bacteria, and the release characteristic of CP@CDs could affect toward *S. aureus > P. aeruginosa > E. coli* under slow drug release of CP to reach the maximum value at ~690 µg mL^−1^, ~2650 µg mL^−1^ and ~3080 µg mL^−1^ from CP@CDs-100, CP@CDs-200 and CP@CDs-400 within 48 h at pH 7.4, respectively.

**Table 3.** Antimicrobial activity test of CDs, and CP@CDs at different loading ratios.

Samples	Microorganisms	Antimicrobial susceptibility test	
		MICs (mg mL^−1^)	MLCs (mg mL^−1^)
CDs-280C1F^a^	*Staphylococcus aureus* ATCC 25923	>250.00	>250.00
	*Pseudomonas aeruginosa* ATCC 27853	>250.00	>250.00
	*Escherichia coli* ATCC 25922	>250.00	>250.00
CP@CDs-100^a^	*Staphylococcus aureus* ATCC 25923	7.81	>250.00
	*Pseudomonas aeruginosa* ATCC 27853	15.62	125.00
	*Escherichia coli* ATCC 25922	0.12	125.00
CP@CDs-200^a^	*Staphylococcus aureus* ATCC 25923	31.25	>250.00
	*Pseudomonas aeruginosa* ATCC 27853	62.50	125.00
	*Escherichia coli* ATCC 25922	0.12	31.25
CP@CDs-400^a^	*Staphylococcus aureus* ATCC 25923	31.25	>250.00
	*Pseudomonas aeruginosa* ATCC 27853	15.62	>250.00
	*Escherichia coli* ATCC 25922	0.12	125.00
CP^b^(Positive control)	*Staphylococcus aureus* ATCC 25923	<0.48 × 10^−3^	0.49
	*Pseudomonas aeruginosa* ATCC 27853	<0.48 × 10^−3^	0.98
	*Escherichia coli* ATCC 25922	<0.48 × 10^−3^	0.95 × 10^−3^

CDs was synthesized from sucrose solution (5%w/v) in a continuous flow microreactor system at 1 mL min^−1^, 280°C. ^a^concentration of 250 mg mL^−1^ and ^b^concentration of 500 mg mL^−1^.

### 3.4. Cytotoxicity study of carbon dots and CP@CDs on L-929 cells

**Figure 15.**
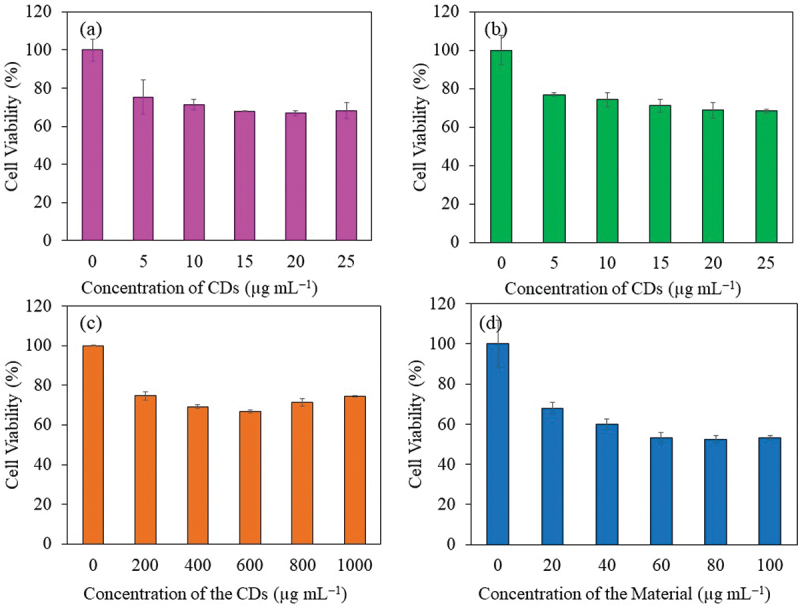
Viability of L-929 cells using MTT assay; (a) S-CDs200C9H, (b) S-CDs200C12H, (c) CDs-280C1F and (d) CP@CDs-200 from CP loaded on CDs-280C1F.

Cytotoxicity study of sucrose derived CDs from batch hydrothermal (S-CDs200C9H and S-CDs200C12H) and continuous flow reactor system synthesis at 280°C and a flow rate of 1 mL min^−1^ (CDs-280C1F) as well as CP-loaded CDs from CDs-280C1F (CP@CDs-200) on mouse fibroblast cell line, L-929 (also known as ATCC # CCL-1) was investigated as illustrated in Figure 15. From the results, S-CDs synthesized from sucrose from both batch and continuous processes showed positive effect on L-929 cell viability. Cell viability was higher than 67% although concentration of CDs was as high as 1000 µg mL^−1^ as illustrated in Figure 15(a–c). However, the L-929 cell viability was reduced from 100% to 53% when tested with CP-loaded CDs-280C1F (CP@CD-200 sample) at a concentration of 60 µg mL^−1^. An increase of CP@CDs-200 concentration from 60 to 100 µg mL^−1^ did not gave further cell viability reduction of L-929 cells as demonstrated in Figure 15(d). To sum up, CDs-280C1F from the continuous production in a flow reactor system at 280°C and flow rate of 1 mL min^−1^ can be used as nanocarrier *in vitro* without toxicity until 1000 µg mL^−1^ while CP@CDs-200 can be used at only 60 µg mL^−1^ to maintain the lowest toxicity not less than 53% cell viability toward L-929 fibroblast cells. The results agreed with minimal inhibitory concentration (MIC) of CP (positive control) in Table 3 which inhibited *S. aureus*, *P. aeruginosa*, and *E. coli* at very low concentration at <0.48 µg mL^−1^ (Table 3). For CP@CDs-200 that released lower rate of CP at the initial state, the inhibitory effect was found for *E. coli > S. aureus > P. aeruginosa*.

## 4. Conclusion

CDs were successfully synthesized from xylose, glucose, sucrose and table sugar using a batch hydrothermal method. Sucrose precursor gave CDs having highest QY (175.48%) and average diameter (~6.8 ± 1.1 nm) when synthesized at 220°C for 9 h. Since S-CDs from sucrose exhibited most excellent efficiency in all aspects, namely, high QY, highest mass yield, supreme crystallinity through Raman spectra, greatest stability due to highest zeta potential and utmost carbon content from XPS analysis; therefore, sucrose was chosen as a precursor for CDs synthesis via a continuous hydrothermal process. The most suitable condition for CDs synthesis from sucrose in a continuous flow reactor system was at 280°C when the flow rate was 1 mL min^−1^. The overall synthesis time for CDs was markedly reduced from 9 h to around 4.4 min, and the high %QY of 53% was achieved by using a continuous flow reactor system. The continuous process produced CDs at 6.6 mg min^−1^ capacity which was significantly higher than the batch hydrothermal process (0.19 mg min^−1^). The synthesized CDs were conjugated effectively with CP, an antimicrobial drug, and an efficiently pH responsive release of CP was achieved. To sum up, CDs potentially serve as an excellent nanocarrier with good functionality, FL properties and noticeably high biocompatibility for further nanomedicine and nanotherapeutic combined with bioimaging applications.

## Supplementary Material

Supplemental Material

## References

[1] Kang C, Huang Y, Yang H, et al. A review of carbon dots produced from biomass wastes. Nanomaterials (Basel). 2020 Nov 23;10(11):2316. doi: 10.3390/nano10112316 33238367 PMC7700468

[2] Yoo D, Park Y, Cheon B, et al. Carbon dots as an effective fluorescent sensing platform for metal ion detection. Nanoscale Res Lett. 2019;14(1):1–23. doi: 10.1186/s11671-019-3088-6 31410663 PMC6692426

[3] Pham-Truong T-N, Petenzi T, Ranjan C, et al. Microwave assisted synthesis of carbon dots in ionic liquid as metal free catalyst for highly selective production of hydrogen peroxide. Carbon. 2018;130:544–552. doi: 10.1016/j.carbon.2018.01.070

[4] Hu C, Li M, Qiu J, et al. Design and fabrication of carbon dots for energy conversion and storage. Chem Soc Rev. 2019;48(8):2315–2337. doi: 10.1039/C8CS00750K 30882820

[5] Tuerhong M, Xu Y, Yin X-B. Review on carbon dots and their applications. Chin J Anal Chem. 2017;45(1):139–150. doi: 10.1016/S1872-2040(16)60990-8

[6] Kong T, Hao L, Wei Y, et al. Doxorubicin conjugated carbon dots as a drug delivery system for human breast cancer therapy. Cell Prolif. 2018 Oct;51(5):e12488. doi: 10.1111/cpr.12488 30039515 PMC6528846

[7] Thakur M, Pandey S, Mewada A, et al. Antibiotic conjugated fluorescent carbon dots as a theranostic agent for controlled drug release, bioimaging, and enhanced antimicrobial activity. J Drug Deliv. 2014;2014:1–9. doi: 10.1155/2014/282193 PMC3976943 24744921

[8] Cui X, Zhu L, Wu J, et al. A fluorescent biosensor based on carbon dots-labeled oligodeoxyribonucleotide and graphene oxide for mercury (II) detection. Biosens Bioelectron. 2015;63:506–512. doi: 10.1016/j.bios.2014.07.085 25137567

[9] Park SY, Lee HU, Park ES, et al. Photoluminescent green carbon nanodots from food-waste-derived sources: large-scale synthesis, properties, and biomedical applications. ACS Appl Mater Interfaces. 2014 Dec 3;6(5):3365–3370. doi: 10.1021/am500159p 24512145

[10] Jiang K, Wang Y, Gao X, et al. Facile, quick, and gram‐scale synthesis of ultralong‐lifetime room‐temperature‐phosphorescent carbon dots by microwave irradiation. Angew Chem Int Ed. 2018;57(21):6216–6220. doi: 10.1002/anie.201802441 29637675

[11] Wang P, Liu C, Tang W, et al. Molecular glue strategy: large-scale conversion of clustering-induced emission luminogen to carbon dots. ACS Appl Mater Inter. 2019;11(21):19301–19307. doi: 10.1021/acsami.8b22605 31062574

[12] Du XY, Wang CF, Wu G, et al. The rapid and large‐scale production of carbon quantum dots and their integration with polymers. Angew Chem Int Ed. 2021;60(16):8585–8595. doi: 10.1002/anie.202004109 32410267

[13] Baragau I-A, Power NP, Morgan DJ, et al. Efficient continuous hydrothermal flow synthesis of carbon quantum dots from a targeted biomass precursor for on–off metal ions nanosensing. ACS Sustainable Chem Eng. 2021;9(6):2559–2569. doi: 10.1021/acssuschemeng.0c08594

[14] Baragau I-A, Lu Z, Power NP, et al. Continuous hydrothermal flow synthesis of S-functionalised carbon quantum dots for enhanced oil recovery. Chem Eng J. 2021;405:126631. doi: 10.1016/j.cej.2020.126631

[15] Shao M, Yu Q, Jing N, et al. Continuous synthesis of carbon dots with full spectrum fluorescence and the mechanism of their multiple color emission. Lab Chip. 2019 Dec 7;19(23):3974–3978. doi: 10.1039/C9LC00683D 31659359

[16] Rao L, Tang Y, Li Z, et al. Efficient synthesis of highly fluorescent carbon dots by microreactor method and their application in Fe(3+) ion detection. Mater Sci Eng C Mater Biol Appl. 2017 Dec 1;81:213–223. 28887967 10.1016/j.msec.2017.07.046

[17] Pedro S-D, Salinas-Castillo A, Ariza-Avidad M, et al. Microsystem-assisted synthesis of carbon dots with fluorescent and colorimetric properties for pH detection. Nanoscale. 2014;6(11):6018–6024. doi: 10.1039/C4NR00573B 24777567

[18] Li L, Wang X, Fu Z, et al. One-step hydrothermal synthesis of nitrogen- and sulfur-co-doped carbon dots from ginkgo leaves and application in biology. Mater Lett. 2017 Jun 1;196:300–303.

[19] Sangjan A, Boonsith S, Sansanaphongpricha K, et al. Facile preparation of aqueous-soluble fluorescent polyethylene glycol functionalized carbon dots from palm waste by one-pot hydrothermal carbonization for colon cancer nanotheranostics. Sci Rep. 2022 Jun 22;12(1):10550. doi: 10.1038/s41598-022-14704-x 35732805 PMC9217983

[20] Saengsrichan A, Saikate C, Silasana P, et al. The role of N and S doping on photoluminescent characteristics of carbon dots from palm bunches for fluorimetric sensing of Fe3+ ion. Int J Mol Sci. 2022;23(9):5001. doi: 10.3390/ijms23095001 35563393 PMC9100793

[21] Saengsrichan A, Khemthong P, Wanmolee W, et al. Platinum/carbon dots nanocomposites from palm bunch hydrothermal synthesis as highly efficient peroxidase mimics for ultra-low H2O2 sensing platform through dual mode of colorimetric and fluorescent detection. Anal Chim Acta. 2022;1230:340368. doi: 10.1016/j.aca.2022.340368 36192059

[22] Wei L, Ma Y, Shi X, et al. Living cell intracellular temperature imaging with biocompatible dye-conjugated carbon dots. J Mater Chem B. 2017;5(18):3383–3390. doi: 10.1039/C7TB00309A 32264404

[23] Sudolská M, Dubecký M, Sarkar S, et al. Nature of absorption bands in oxygen-functionalized graphitic carbon dots. J Phys Chem C. 2015 Jun 11;119(23):13369–13373. doi: 10.1021/acs.jpcc.5b04080

[24] Mansuriya BD, Altintas Z. Carbon dots: classification, properties, synthesis, characterization, and applications in Health care—an updated review (2018–2021). Nanomaterials (Basel). 2021 Sep 27;11(10):2525. doi: 10.3390/nano11102525 PMC8541690 34684966

[25] Tauc J, Menth A. States in the gap. J Non-Cryst Solids. 1972 June 1;8-10:569–585. doi: 10.1016/0022-3093(72)90194-9.

[26] Akhavan O, Tohidi H, Moshfegh AZ. Synthesis and electrochromic study of sol–gel cuprous oxide nanoparticles accumulated on silica thin film. Thin Solid Films. 2009 Oct 30;517(24):6700–6706.

[27] Saadati M, Akhavan O, Fazli H. Single-layer MoS2-MoO3-x heterojunction nanosheets with simultaneous photoluminescence and co-photocatalytic features. Catalysts. 2021;11(12):1445. doi: 10.3390/catal11121445

[28] Jumardin J, Maddu A, Santoso K, et al. Synthesis of carbon dots (CDS) and determination of optical gap energy with Tauc plot method. Jambura Phys J. 2021 Nov 1;3:73–86.

[29] Sakdaronnarong C, Sangjan A, Boonsith S, et al. Recent developments in synthesis and photocatalytic applications of carbon dots. Catalysts. 2020;10(3):320. doi: 10.3390/catal10030320

[30] Ding H, Wei J-S, Zhang P, et al. Solvent-controlled synthesis of highly luminescent carbon dots with a wide color gamut and narrowed emission peak widths. Small. 2018;14(22):1800612. doi: 10.1002/smll.201800612 29709104

[31] Zhao B, Ma H, Zheng M, et al. Narrow-bandwidth emissive carbon dots: a rising star in the fluorescent material family. Carbon Energy. 2022;4(1):88–114. doi: 10.1002/cey2.175

[32] Chamnankid B, Ratanatawanate C, Faungnawakij K. Conversion of xylose to levulinic acid over modified acid functions of alkaline-treated zeolite Y in hot-compressed water. Chem Eng J. 2014 Dec 15;258:341–347. doi: 10.1016/j.cej.2014.07.036.

[33] Tan-Soetedjo JNM, van de Bovenkamp HH, Abdilla RM, et al. Experimental and kinetic modeling studies on the conversion of sucrose to levulinic acid and 5-hydroxymethylfurfural using sulfuric acid in water. Ind Eng Chem Res. 2017 Nov 15;56(45):13228–13239. doi: 10.1021/acs.iecr.7b01611 29170598 PMC5695899

[34] Kutrakul N, Liu A, Ratchahat S, et al. Highly selective catalytic conversion of raw sugar and sugarcane bagasse to lactic acid over YbCl3, ErCl3, and CeCl3 Lewis acid catalysts without alkaline in a hot-compressed water reaction system. Chem Eng Res Des. 2022 Nov 1;187:549–569.

[35] Voß D, Ponce S, Wesinger S, et al. Combining autoclave and LCWM reactor studies to shed light on the kinetics of glucose oxidation catalyzed by doped molybdenum-based heteropoly acids. RSC Adv. 2019;9(50):29347–29356. doi: 10.1039/C9RA05544D 35528392 PMC9071830

[36] Papaioannou N, Marinovic A, Yoshizawa N, et al. Structure and solvents effects on the optical properties of sugar-derived carbon nanodots. Sci Rep. 2018 Apr 26;8(1):6559. doi: 10.1038/s41598-018-25012-8 29700398 PMC5920085

[37] Wang S, Yang D-S, Yang F. Nitrogen-induced shift of photoluminescence from green to blue emission for xylose-derived carbon dots. Nano Ex. 2020 Jul;1(2):020018. Related Information: CHORUS Timestamp: 2021-11-26 13:30:55. 2020:Medium: X; Size: Article No. 020018. doi: 10.1088/2632-959X/aba771

[38] Reckmeier CJ, Schneider J, Susha AS, et al. Luminescent colloidal carbon dots: optical properties and effects of doping [invited]. Opt Express. 2016 Jan 25;24(2):A312–A340. doi: 10.1364/OE.24.00A312 26832584

[39] Joseph E, Singhvi G. Chapter 4 – multifunctional nanocrystals for cancer therapy: a potential nanocarrier. In: Grumezescu A, editor. Nanomaterials for drug delivery and therapy. Norwich (NY): William Andrew Publishing; 2019. p. 91–116.

[40] Yahaya Pudza M, Zainal Abidin Z, Abdul Rashid S, et al. Eco-friendly sustainable fluorescent carbon dots for the adsorption of heavy metal ions in aqueous environment. Nanomaterials. 2020;10(2):315. doi: 10.3390/nano10020315 32059384 PMC7075143

[41] Yang X, Sui L, Wang B, et al. Red-emitting, self-oxidizing carbon dots for the preparation of white LEDs with super-high color rendering index. Sci China Chem. 2021 Sep 1;64(9):1547–1553. doi: 10.1007/s11426-021-1033-6

[42] Yoo HJ, Kwak BE, Kim DH. Competition of the roles of π-conjugated domain between emission center and quenching origin in the photoluminescence of carbon dots depending on the interparticle separation. Carbon. 2021 Oct 15;183:560–570. doi: 10.1016/j.carbon.2021.07.054

[43] Shabbir H, Csapó E, Wojnicki M. Carbon quantum dots: the role of surface functional groups and proposed mechanisms for metal ion sensing. Inorganics. 2023;11(6):262. doi: 10.3390/inorganics11060262

[44] de Medeiros TV, Manioudakis J, Noun F, et al. Microwave-assisted synthesis of carbon dots and their applications. J Mater Chem C. 2019;7(24):7175–7195. doi: 10.1039/C9TC01640F

[45] Yao H, Xu Z, Cheng M, et al. Catalytic conversion of formic acid to methanol with Cu and Al under hydrothermal conditions. BioResources. 2012;7(1):0972–0983. doi: 10.15376/biores.7.1.972-983

[46] González Calderón JA, Contreras López D, Pérez E, et al. Polysiloxanes as polymer matrices in biomedical engineering: their interesting properties as the reason for the use in medical sciences. Polym Bull. 2020;77(5):2749–2817. doi: 10.1007/s00289-019-02869-x

[47] Gotor V, Gotor‐Fernández V, Busto E, editors. 7.6 Hydrolysis and reverse hydrolysis: hydrolysis and formation of amides. 2012. doi: 10.1016/B978-0-08-095167-6.00707-2

[48] Zhang W, Hu W, Wen CK. Ethylene preparation and its application to physiological experiments. Plant Signal Behav. 2010 Apr;5(4):453–457. doi: 10.4161/psb.5.4.10875 20118671 PMC2958598

[49] Xu S, Wang L, Chu W, et al. Influence of Pd precursors on the catalytic performance of Pd–H4SiW12O40/SiO2 in the direct oxidation of ethylene to acetic acid. J Mol Catal A Chem. 2009;310(1–2):138–143. doi: 10.1016/j.molcata.2009.06.008

[50] Ansi VA, Sreelakshmi P, Poovathinthodiyil R, et al. Table sugar derived carbon dot—a promising green reducing agent. Mater Res Bull. 2021 Jul 1;139:111284.

[51] Katea SN, Westin G. Carbothermal nitridation of solution synthesised ZrO2–carbon nanocomposites; phase-development from precursor to nitride. Ceram Int. 2021;47(8):10828–10847. doi: 10.1016/j.ceramint.2020.12.200

[52] Pierna J, Abbas O, Dardenne P, et al. Discrimination of Corsican honey by FT-Raman spectroscopy and chemometrics. Biotechnologie, Agronomie, Société et Environnement. 2011;15:75–84.

[53] Gameiro Dos Santos J, Figueirinhas R, Liberal JP, et al. On ciprofloxacin concentration in chronic rhinosinusitis. Acta Otorrinolaringol Esp (Engl Ed). 2018 Jan–Feb;69(1):35–41. doi: 10.1016/j.otorri.2017.06.008 28859993

[54] Zulfiqar H, Zafar A, Rasheed MN, et al. Synthesis of silver nanoparticles using Fagonia cretica and their antimicrobial activities. Nanoscale Adv. 2019;1(5):1707–1713. doi: 10.1039/C8NA00343B 36134229 PMC9473189

[55] Hajidariyor T, Nuntawad N, Somsaen P, et al. Cryo-induced Cellulose-based Nanogel from Elaeis guineensis for antibiotic delivery platform. Int J Mol Sci. 2023 Jan 8;24(2):1230. doi: 10.3390/ijms24021230 36674748 PMC9866051

[56] Yang S, Mu L, Feng R, et al. Selection of internal standards for quantitative matrix-assisted laser desorption/ionization mass spectrometric analysis based on correlation coefficients. ACS Omega. 2019;4(5):8249–8254. doi: 10.1021/acsomega.9b00566 31459912 PMC6648383

[57] Sreedharan SM, Singh R. Ciprofloxacin functionalized biogenic gold nanoflowers as nanoantibiotics against pathogenic bacterial strains. Int J Nanomed. 2019;14: 9905. doi: 10.2147/IJN.S224488 PMC6927271 31908448

[58] Kooti M, Sedeh AN, Motamedi H, et al. Magnetic graphene oxide inlaid with silver nanoparticles as antibacterial and drug delivery composite. Appl Microbiol Biotechnol. 2018;102(8):3607–3621. doi: 10.1007/s00253-018-8880-1 29511845

[59] Mashhadizadeh MH, Amoli-Diva M. Drug-carrying amino silane coated magnetic nanoparticles as potential vehicles for delivery of antibiotics. J Nanomed Nanotechnol. 2012;3(4):1. doi: 10.4172/2157-7439.1000139

