# Supplementary material for: Scaling-up of carbon dots hydrothermal synthesis from sugars in a continuous flow microreactor system for biomedical application as in vitro antimicrobial drug nanocarrier
Source: Sci Technol Adv Mater. 2023 Oct 17;24(1):2260298. doi: 10.1080/14686996.2023.2260298 (PMC10583617; doi:10.1080/14686996.2023.2260298)
Supplement: Supplemental Material [file TSTA_A_2260298_SM2574.docx]

**Electronic Supporting Information**

**Scaling-up of carbon dots hydrothermal synthesis from sugars in a continuous flow microreactor system for biomedical application as *in vitro* antimicrobial drug nano-carrier**

Siriboon Supajaruwong^1,#^, Sirawich Porahong^1, #^, Agung Wibowo^1^, Yu-Sheng Yu^2^,

Mohd Jahir Khan^1^, Pisut Pongchaikul^3^, Pattaraporn Posoknistakul^1^, Navadol Laosiripojana^4^,

Kevin C.-W. Wu^2,5,*^, Chularat Sakdaronnarong^1,**^

^1^ Department of Chemical Engineering, Faculty of Engineering, Mahidol University, Nakhon Pathom, 73170, Thailand

^2^ Department of Chemical Engineering, National Taiwan University, No.1, Sec.4 Roosevelt Road, Taipei, 10617 Taiwan

^3^ Chakri Naruebodindra Medical Institute, Faculty of Medicine Ramathibodi Hospital, Mahidol University, Samut Prakarn, 10540 Thailand

^4^ The Joint Graduate School of Energy and Environment, King Mongkut's University of Technology Thonburi, 126 Pracha Uthit Road, Bang Mot, Thung Khru, Bangkok 10140 Thailand

^5^ National Health Research Institutes, Institute of Biomedical Engineering and Nanomedicine, Miaoli, 35053, Taiwan

* Corresponding author: kevinwu@ntu.edu.tw

** Corresponding author: chularat.sak@mahidol.ac.th;

^#^ The authors contributed equally as co-first authors

Tel: +662-8892138 ext. 6101-2, Fax: +662-4419731


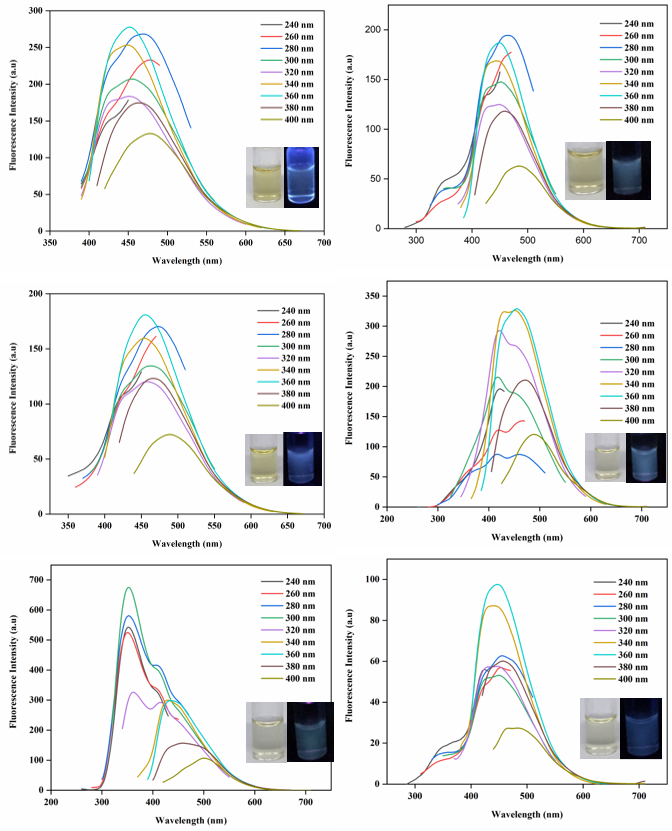


f)

e)

d)

c)

b)

a)

**Figure S1.** The fluorescence emission spectra of X-CDs synthesized via a batch hydrothermal at vary temperature and time (a) 200C6H (b) 220C6H (c) 200C9H (d) 220C9H (e) 200C12H (f) 220C12H at various excitation wavelengths from 240 nm to 400 nm with an interval of 20 nm.


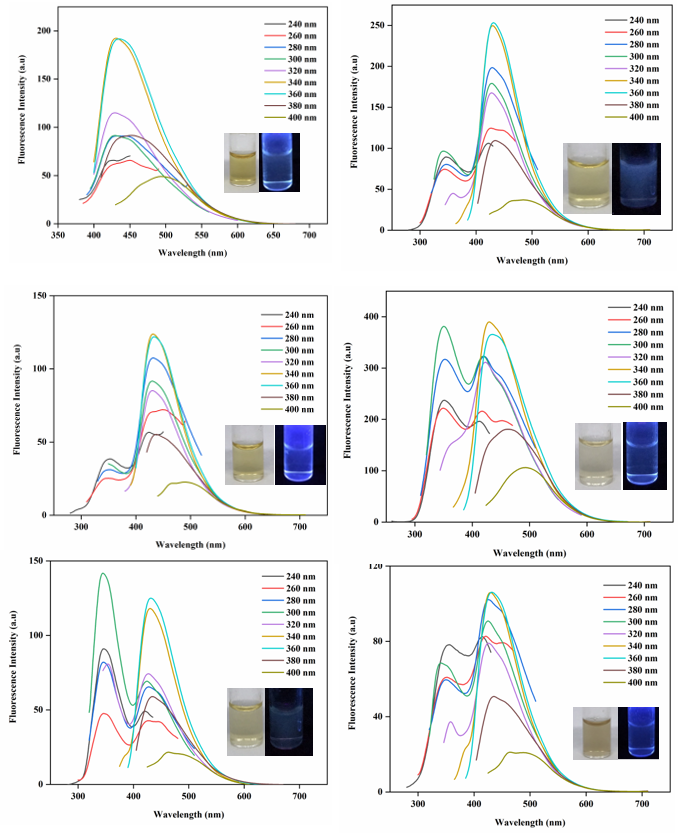


f)

e)

d)

c)

b)

a)

**Figure S2.** The fluorescence emission spectra of G-CDs synthesized via a batch hydrothermal at vary temperature and time (a) 200C6H (b) 220C6H (c) 200C9H (d) 220C9H (e) 200C12H (f) 220C12H at various excitation wavelengths from 240 nm to 400 nm with an interval of 20 nm.


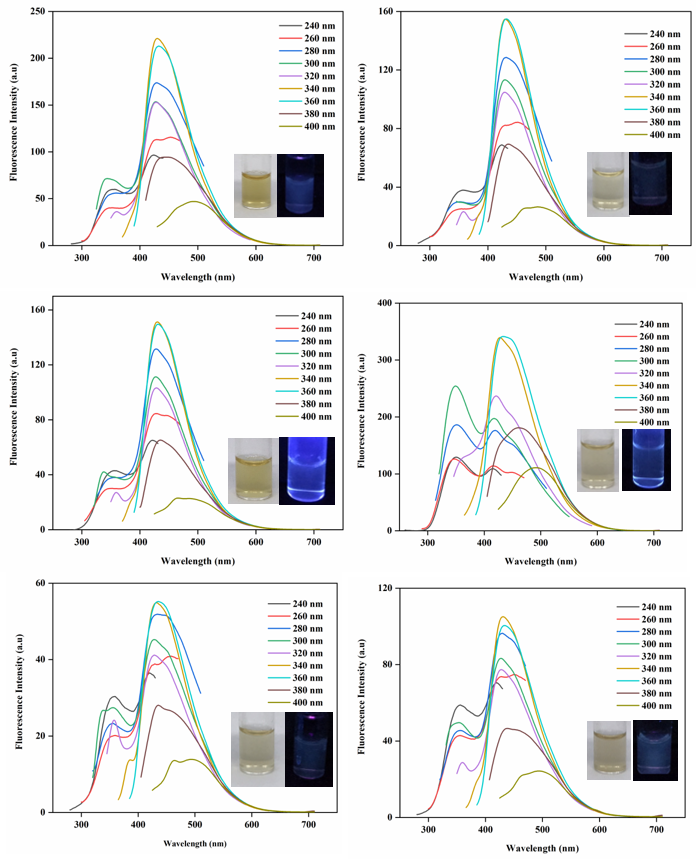


f)

e)

d)

c)

b)

a)

**Figure S3.** The fluorescence emission spectra of T-CDs synthesized via a batch hydrothermal at vary temperature and time (a) 200C6H (b) 220C6H (c) 200C9H (d) 220C9H (e) 200C12H (f) 220C12H at various excitation wavelengths from 240 nm to 400 nm with an interval of 20 nm.

b)

a)


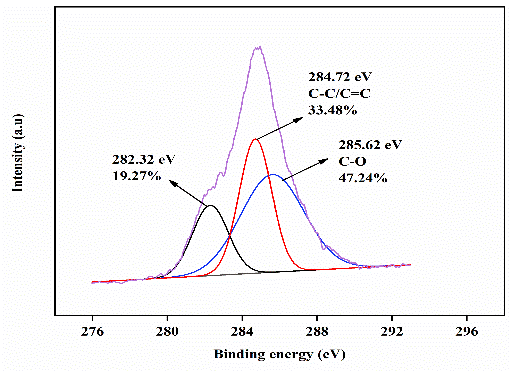

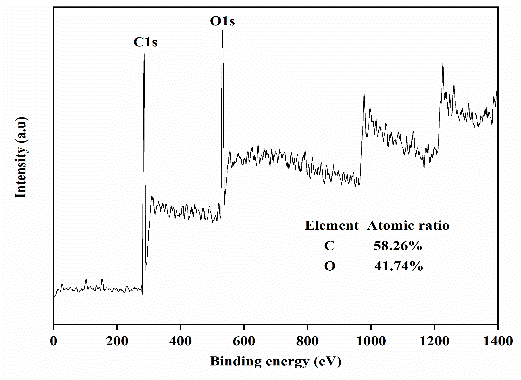

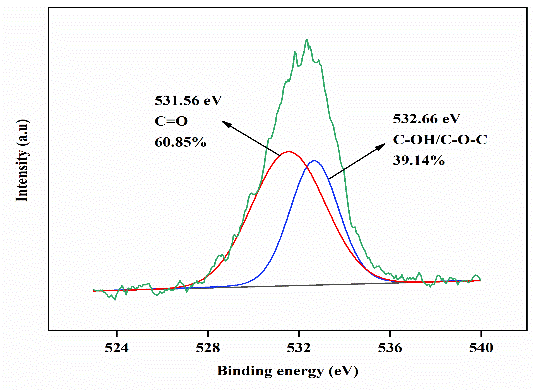

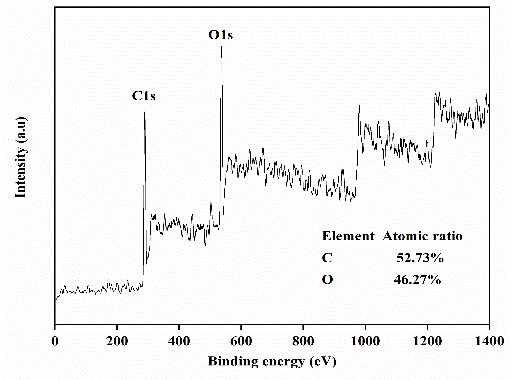

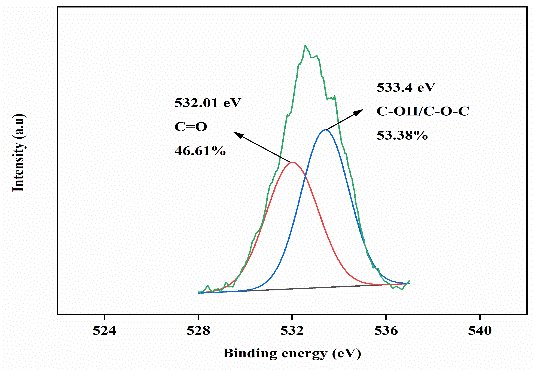

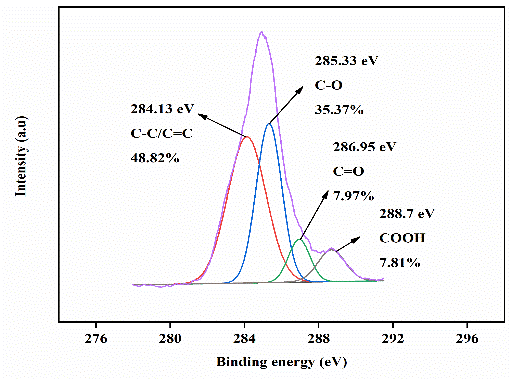


d)

c)

f)

e)

**Figure S4.** The XPS analysis of X-CD (a-b) Survey scan 200 °C and 220 °C, (c-d) C1s 200 °C and 220 °C, (e-f) O1s 200 °C and 220 °C when the synthesis time was 9 h.

b)

a)


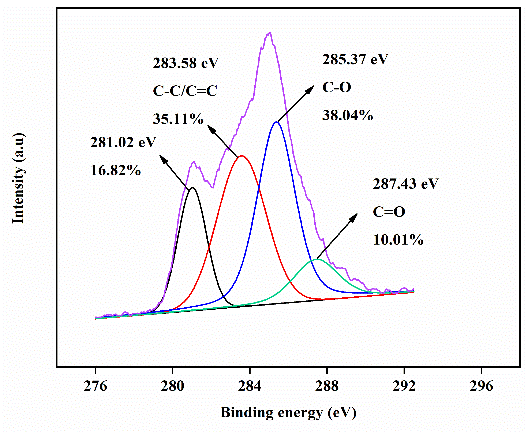

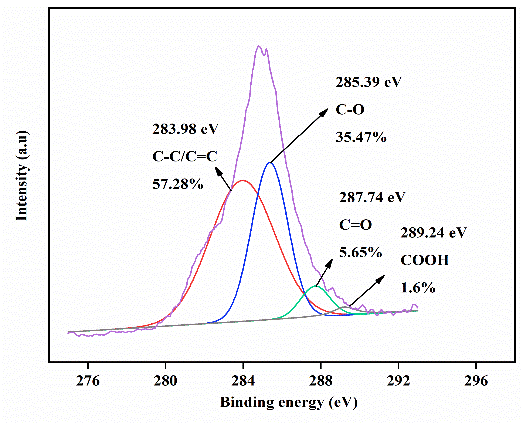

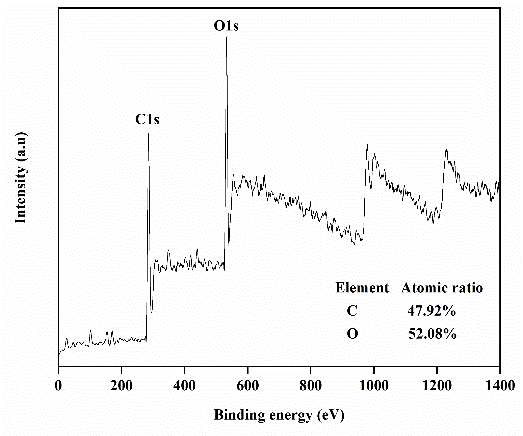

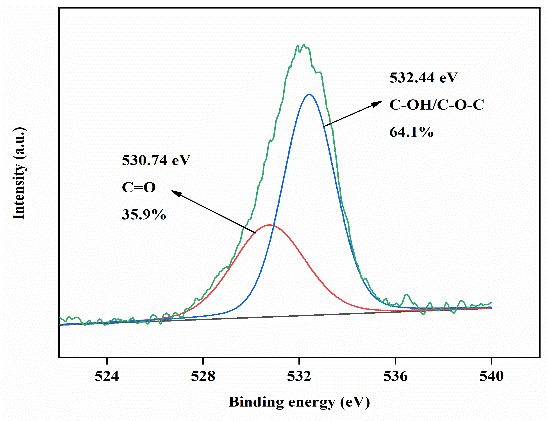

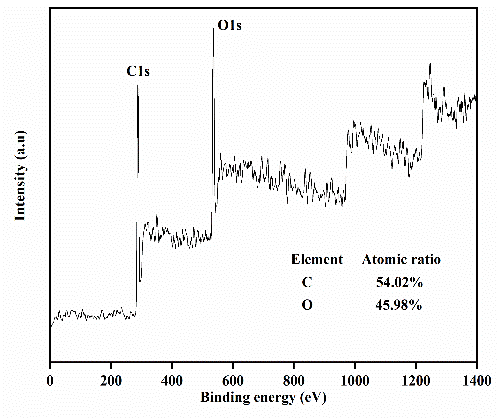

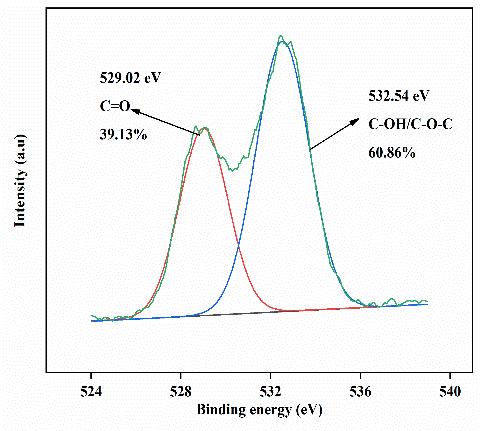


d)

c)

+

e)

f)

**Figure S5.** The XPS analysis of G-CD (a-b) Survey scan 200 °C and 220 °C, (c-d) C1s 200 °C and 220 °C, (e-f) O1s 200 °C and 220 °C when the synthesis time was 9 h.

b)

a)


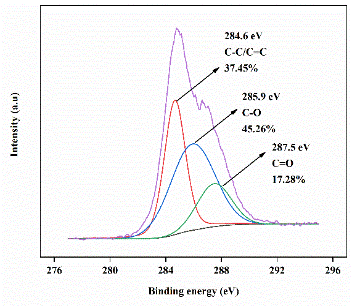

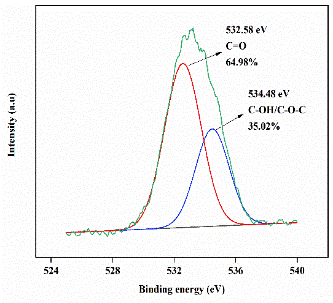

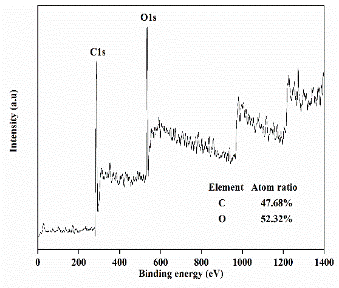

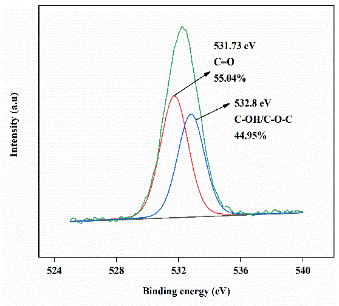

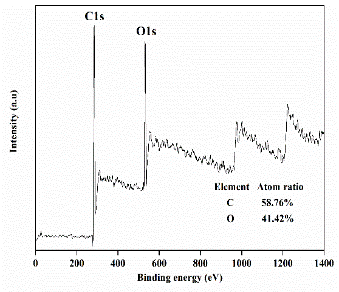

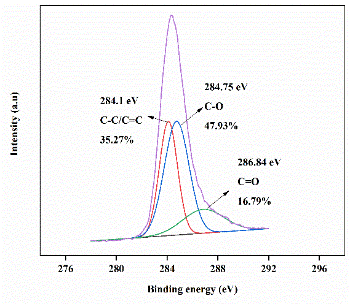


c

e

f

d

b

d)

c)

f)

e)

**Figure S6.** The XPS analysis of T-CD (a-b) Survey scan 200 °C and 220 °C, (c-d) C1s 200 °C and 220 °C, (e-f) O1s 200 °C and 220 °C when the synthesis time was 9 h.


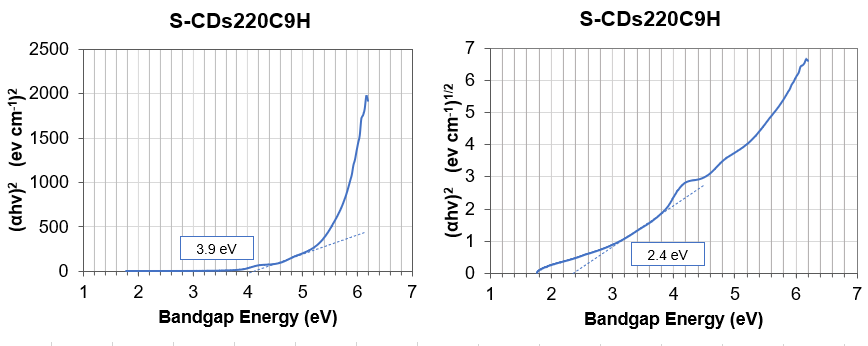


b)

a)

**Figure S7.** Tauc’s plot for (a) direct and (b) indirect band gap energies of S-CDs220C9H. The direct band gap was attributed to the carbon dots domains. The indirect band gap can be originated from heteroatom or heterojunction of multi-layer carbon dots domains. The intercept of dash lines with the horizontal axis defines the value of the band gaps.


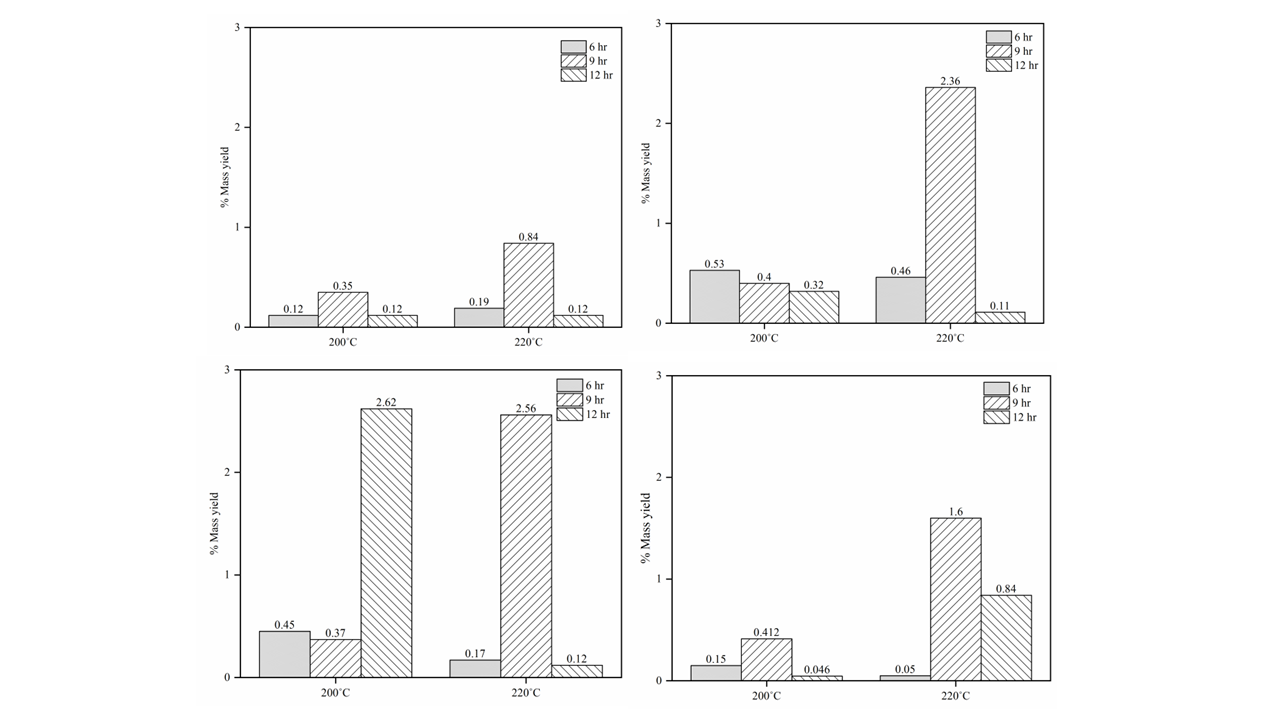


d)

c)

b)

a)

**Figure S8.** The %mass yield of (a) X-CDs, (b) G-CDs, (c) S-CDs, and (d) T-CDs via a batch hydrothermal method at varying temperature and time for CDs synthesis.

**
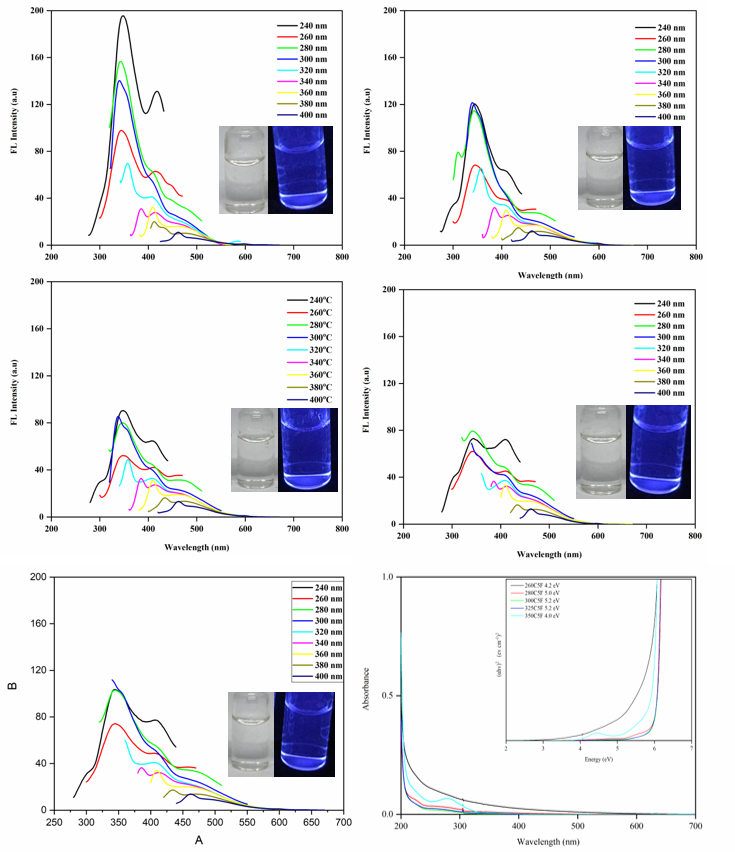
**

f)

e)

d)

c)

b)

a)

**Figure S9.** The fluorescence emission spectra of CDs synthesized via a continuous hydrothermal at a flow rate of 1 mL min^-1^ and vary temperature (a) 260 °C, (b) 280 °C, (c) 300 °C, (d) 325 °C, (e) 350 °C at various excitation wavelengths from 240 nm to 400 nm with an interval of 20 nm. (f) The UV-Visible absorption spectra of the ample of CDs at flow rate 5 mL min^-1^ with a scanned wavelength from 200 – 600 nm; inset shows optical bandgap from Tauc Plot.

**
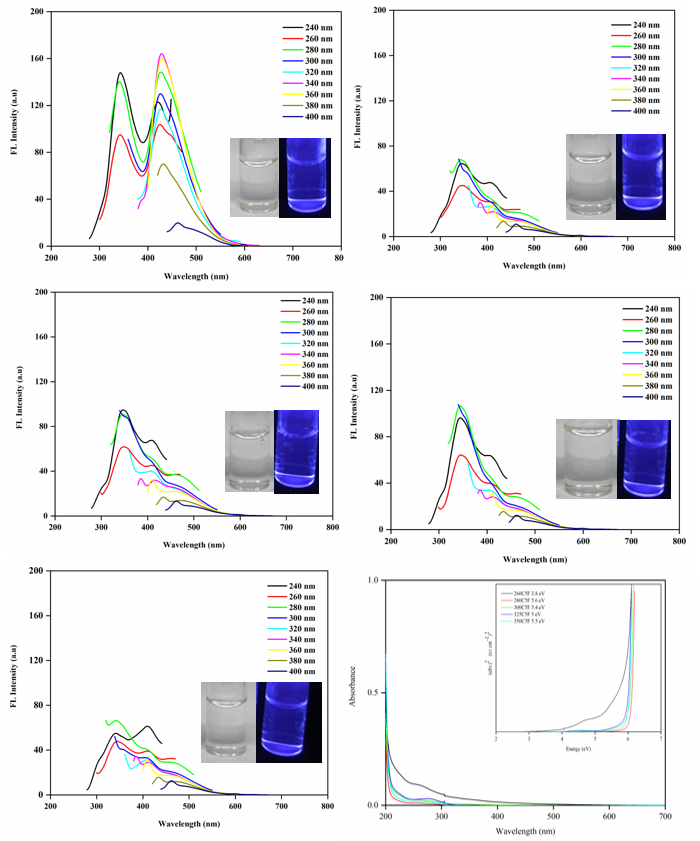
**

f)

e)

d)

c)

b)

a)

**Figure S10.** The fluorescence emission spectra of CDs synthesized via a continuous hydrothermal at a flow rate of 1 mL min^-1^ and vary temperature (a) 260 °C, (b) 280 °C, (c) 300 °C, (d) 325 °C, (e) 350 °C at various excitation wavelengths from 240 nm to 400 nm with an interval of 20 nm. (f) The UV-Visible absorption spectra of the ample of CDs at flow rate 10 mL min^-1^ with a scanned wavelength from 200–600 nm; inset shows optical bandgap from Tauc Plot.


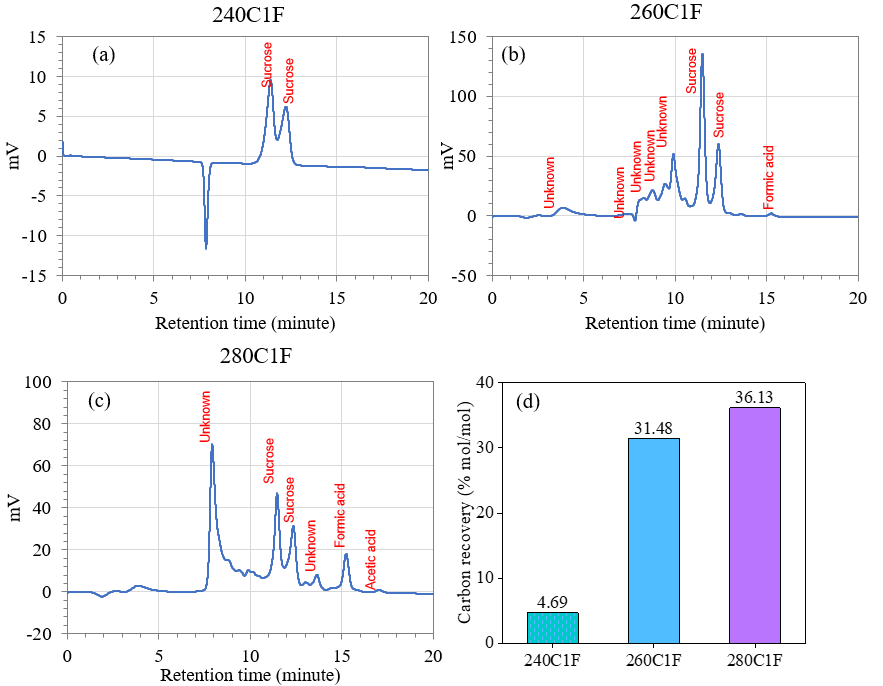


**Figure S11.** HPLC chromatogram of S-CDs synthesized from a continuous flow reactor system at different temperatures and flow rates: (a) 240 °C, 1 mL min^-1^, (b) 260 °C, 1 mL min^-1^, (c) 280 °C, 1 mL min^-1^, and (d) carbon recovery of S-CDs synthesized at 1 mL min^-1^ flow rate.


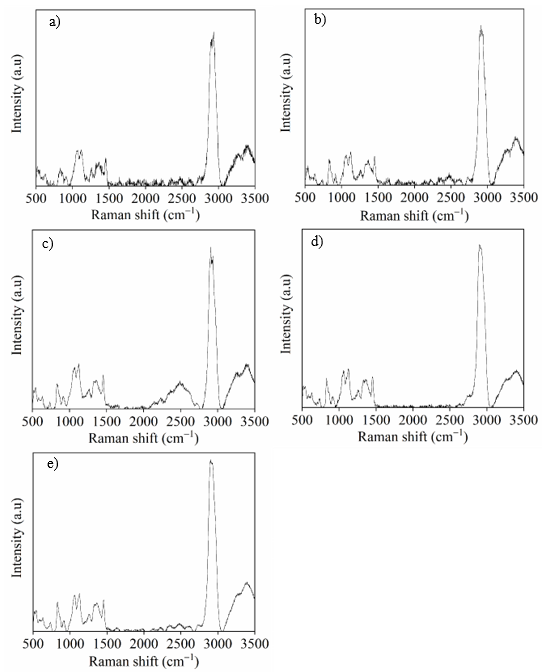


**Figure S12**. Raman spectral profile of S-CDs synthesized via a continuous hydrothermal process by 5 mL min^-1^ at (a) 260 °C, (b) 280 °C, (c) 300 °C, (d) 325 °C, and (e) 350 °C synthesis temperature.


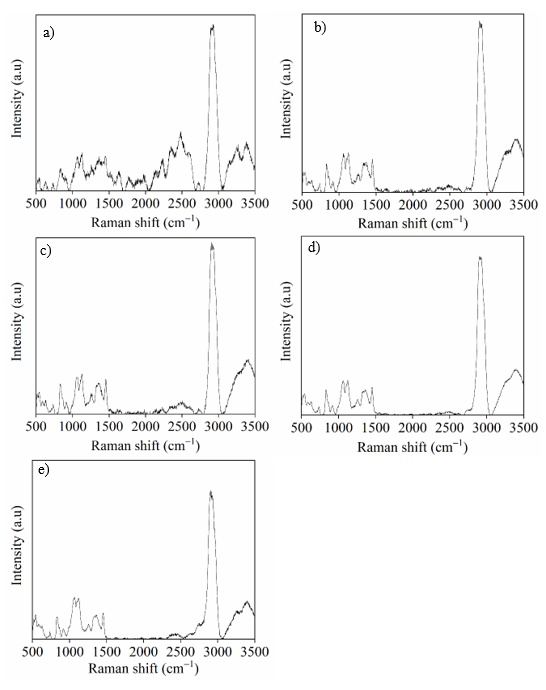


**Figure S13**. Raman spectral profile of S-CDs synthesized via a continuous hydrothermal process by 10 mL min^-1^ at (a) 260 °C, (b) 280 °C, (c) 300 °C, (d) 325 °C, and (e) 350 °C synthesis temperature.


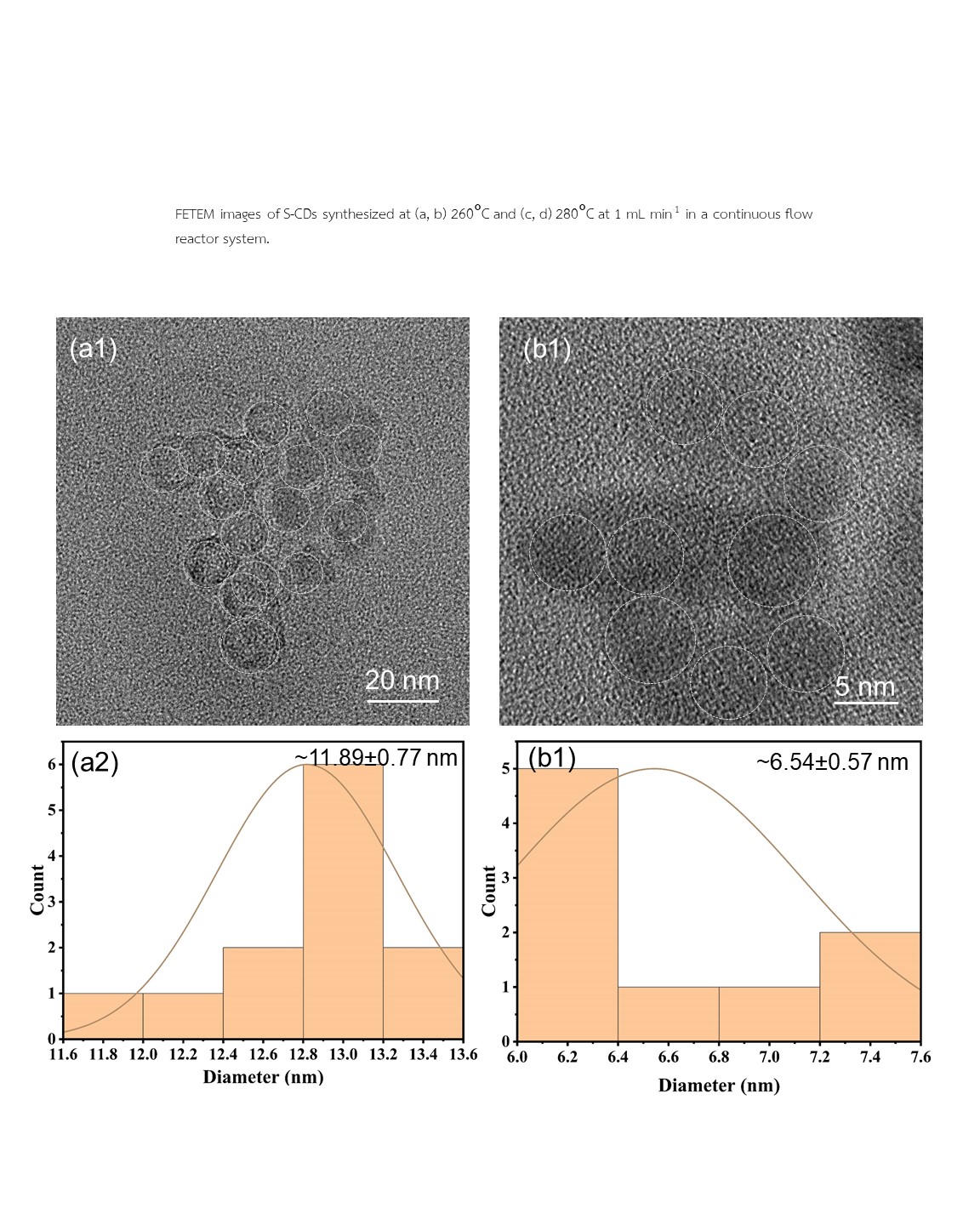


**Figure S14.**  TEM images with particle size distribution of CDs from 1 mL min^-1^ flow reactor system; (a) & (b) 260C1F (260 °C), and (c) & (d) 280C1F (280 °C).

a)

b)

**Figure S15.** (a) Zeta-potential, and (b) %mass yield of S-CDs synthesized via a continuous hydrothermal method at a flow rate of 1 mL min^-1^ and varying temperatures from 240 °C to 280 °C.


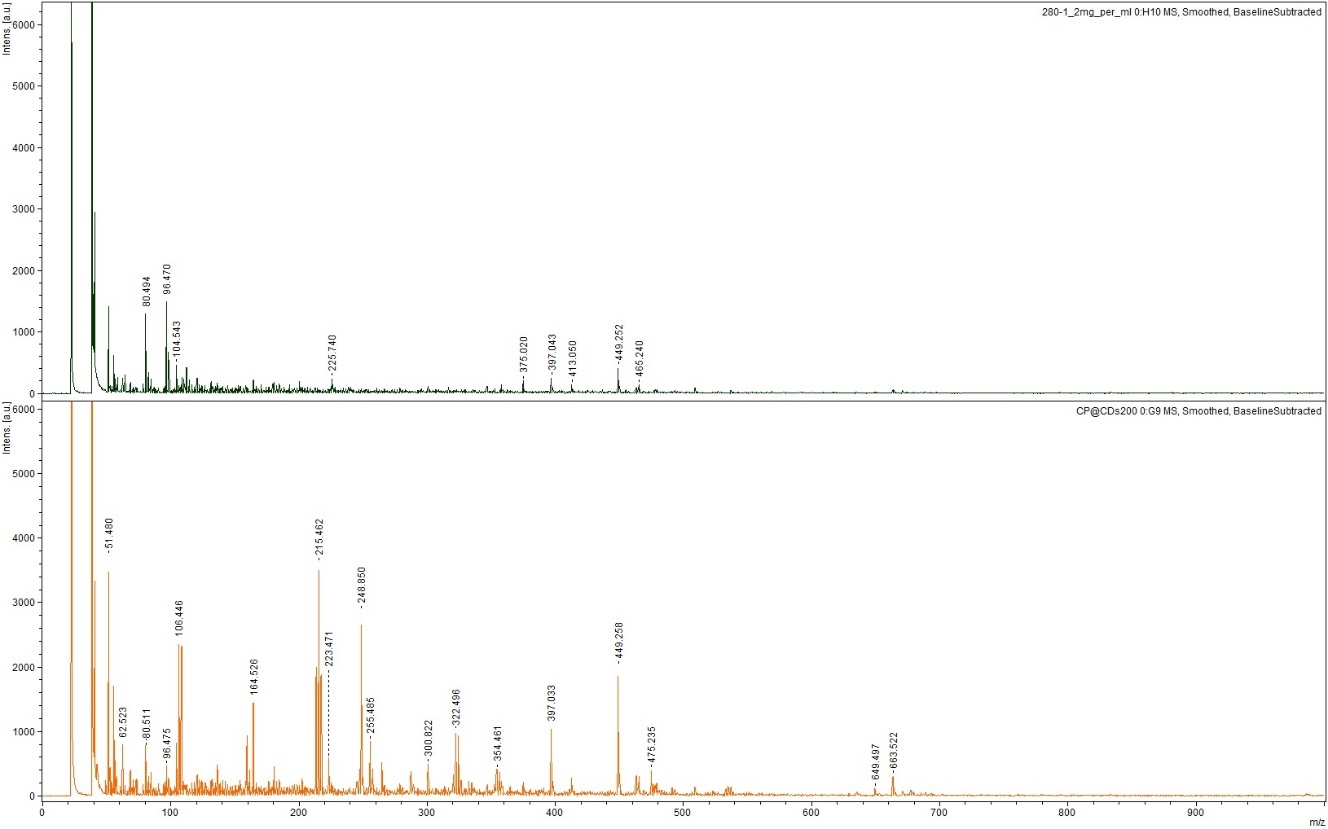


b)

a)

*

*

*

*

**Figure S16.** MALDI-TOF/MS of (a) S-CDs 280C1F synthesized at 280 °C with flow rate of 1 mL min^-1^, and (b) CP loaded on S-CDs 280C1F (CP@CDs-200); Ciprofloxacin quantification relied on the ratio of its parent peak (m/z 215, 248, 322 and 354). (* represents the position of CP)

**Table S1.** LC/MS/MS Orbitrap analysis of intermediate substances from carbon dots synthesis from 5%w/v sucrose solution at 260 °C using 1 mL min^-1^ flow rate in a continuous flow reactor system (Top compounds with most area ≥ 2×10^7^)

| Name | Formula | Molecular Weight | RT [min] | Area (Max.) |
| --- | --- | --- | --- | --- |
| 5-Hydroxymethyl-2-furaldehyde | C6 H6 O3 | 126.0308 | 8.846 | 8.988E+09 |
| Difluorodimethylsilane | C2 H6 F2 Si | 96.02046 | 4.807 | 3.088E+09 |
| Ethephon | C2 H6 Cl O3 P | 143.974 | 22.156 | 2.399E+09 |
| N-Methyl-2-pyrrolidone | C5 H9 N O | 99.06778 | 22.156 | 2.356E+09 |
| 3-hydroxy-3-methylpentanedioic acid | C6 H10 O5 | 144.0413 | 4.833 | 1.135E+09 |
| Dibutyl[(5Z)-6-(butylsulfanyl)-5-decen-5-yl]borane | C22 H45 B S | 352.3318 | 19.619 | 1.124E+09 |
| (1-Aminoethyl)(hydroxy)oxophosphonium | C2 H7 N O2 P | 108.0204 | 9.176 | 854231637 |
| Difluorodimethylsilane | C2 H6 F2 Si | 96.02046 | 5.242 | 764958681 |
| 2H-[1,3,2]Diazaphospholo[4,5-d]pyrimidine | C4 H3 N4 P | 138.0095 | 23.995 | 648520888 |
| Germanecarbonitrile | C H3 Ge N | 102.9476 | 20.664 | 619846653 |
| Pyrogallol | C6 H6 O3 | 126.0308 | 5.528 | 499249270 |
| 1-(7aH-[1,2,3]Triazolo[4,5-b]pyridin-7-yl)-1,2-triazadien-2-ium | C5 H4 N7 | 162.0516 | 5.02 | 472924341 |
| NP-013538 | C12 H16 O8 | 288.0821 | 4.975 | 468071790 |
| O-[{2-[(Diaminomethylene)amino]ethoxy} (hydroxy)phosphoryl]serine | C6 H15 N4 O6 P | 270.0718 | 4.965 | 455827612 |
| Difluorodimethylsilane | C2 H6 F2 Si | 96.02046 | 5.729 | 372036539 |
| N4-(3-chloro-4-fluorophenyl)-6-methylpyrimidine-2,4-diamine hydrochloride | C11 H10 Cl F N4 | 252.0614 | 4.973 | 358517794 |
| D-(+)-Maltose | C12 H22 O11 | 364.095 | 4.701 | 302431395 |
| Acetone | C3 H6 O | 58.04161 | 5.397 | 301767483 |
| Isobutyraldehyde | C4 H8 O | 72.05707 | 4.115 | 272194696 |
| Propionitrile | C3 H5 N | 55.04199 | 24.103 | 239937401 |
| N-[3-(2-methyl-4-pyrimidinyl)phenyl]  -1,3-benzothiazole-2-carboxamide | C19 H14 N4 O S | 346.0847 | 5.407 | 230714792 |
| Isobutyraldehyde | C4 H8 O | 72.05707 | 2.335 | 229262765 |
| NP-013538 | C12 H16 O8 | 310.064 | 8.401 | 221486038 |
| Germanecarbonitrile | C H3 Ge N | 102.9476 | 22.624 | 218663231 |
| Difluorodimethylsilane | C2 H6 F2 Si | 96.02046 | 10.338 | 216545220 |
| Ethyl(dimethyl)phosphine sulfide | C4 H11 P S | 122.0321 | 23.989 | 210038035 |
| 5-Hydroxymethyl-2-furaldehyde | C6 H6 O3 | 126.0308 | 9.315 | 199891592 |
| (3R,4S)-4,6,8-Trihydroxy-7-methoxy  -3-methyl-3,4-dihydro-1H-isochromen-1-one | C11 H12 O6 | 240.0618 | 4.996 | 188605826 |
| Isobutyraldehyde | C4 H8 O | 72.05707 | 5.526 | 177669178 |
| Difluorodimethylsilane | C2 H6 F2 Si | 96.02046 | 6.629 | 174941087 |
| NP-013538 | C12 H16 O8 | 310.064 | 8.94 | 169904995 |
| 4-[(4-Fluorophenyl)ethynyl]-2(5H)-furanone | C12 H7 F O2 | 202.044 | 4.696 | 169090398 |
| 1,4:3,6-Dianhydro-2,5-dideoxy-2-[(ethylcarbamoyl)amino]-5-{[4-(3-fluorophenyl)-2-pyrimidinyl]amino}-L-iditol | C19 H22 F N5 O3 | 387.1707 | 4.813 | 160941272 |
| Isobutyraldehyde | C4 H8 O | 72.05707 | 8.074 | 151596036 |
| Ethyl 4-{[4-amino-6-(3,4-dihydro-2(1H)-isoquinolinyl)-1,3,5-triazin-2-yl]methyl}-1-piperazinecarboxylate | C20 H27 N7 O2 | 397.222 | 21.154 | 130955465 |
| Ethephon | C2 H6 Cl O3 P | 143.974 | 17.381 | 129151898 |
| 3-hydroxy-3-methylpentanedioic acid | C6 H10 O5 | 144.0413 | 5.415 | 128316717 |
| Pyrogallol | C6 H6 O3 | 126.0308 | 10.104 | 125151611 |
| 3-hydroxy-3-methylpentanedioic acid | C6 H10 O5 | 144.0413 | 6.293 | 120352379 |
| 5-Hydroxymethyl-2-furaldehyde | C6 H6 O3 | 126.0308 | 8.408 | 119495233 |
| [Similar to: 3-hydroxy-N-(1-hydroxy-4-methylpentan-2-yl)-5-oxo-6-phenylhexanamide; ΔMass: 2.9089 Da] | C8 H16 N6 O8 | 324.1029 | 5.007 | 115057074 |
| Propionitrile | C3 H5 N | 55.04199 | 24.447 | 108763702 |
| 1-Phenylethan-1-d1-ol | C8 H9 D O | 123.0789 | 23.864 | 108586828 |
| Betaine | C5 H11 N O2 | 117.0781 | 4.826 | 108003312 |
| 2-[3-methyl-2-(methylimino)-4-oxo-1,3-thiazolan-5-yl]acetic acid | C7 H10 N2 O3 S | 202.044 | 4.779 | 105951272 |
| NP-013538 | C12 H16 O8 | 310.064 | 7.646 | 103109992 |
| Difluorodimethylsilane | C2 H6 F2 Si | 96.02046 | 8.842 | 101714133 |
| Methyl palmitate | C17 H34 O2 | 287.2801 | 16.23 | 101569585 |
| 4,4'-[1,2-Ethanediylbis(oxy)]bis(1,2,5-oxadiazol-3-amine) | C6 H8 N6 O4 | 228.0615 | 4.967 | 100821718 |
| Benzothiazole | C7 H5 N S | 135.0134 | 21.548 | 95707053 |
| Acetone | C3 H6 O | 58.04161 | 4.693 | 94664227 |
| Acetamide | C2 H5 N O | 59.03684 | 23.883 | 83958989 |
| 2,4(1H,3H)-Pyrimidinedione-2-14C | C3 [14]C H4 N2 O2 | 114.0309 | 4.92 | 83655698 |
| Benzothiazole | C7 H5 N S | 135.0134 | 23.336 | 82398921 |
| Methyl palmitate | C17 H34 O2 | 287.2801 | 16.384 | 81537884 |
| Benzothiazole | C7 H5 N S | 135.0134 | 20.448 | 80464253 |
| Acetone | C3 H6 O | 58.04161 | 9.193 | 78408201 |
| 1-[2,6-dichloro-4-(trifluoromethyl)phenyl]-3-methyl-1H-pyrazole-4,5-dione 4-(N-phenylhydrazone) | C17 H11 Cl2 F3 N4 O | 414.0319 | 21.246 | 78198056 |
| O,O-Dipropyl (6-amino-4-oxo-1,4-dihydro-2-pyrimidinyl)phosphoramidothioate | C10 H19 N4 O3 P S | 306.0926 | 5.035 | 75512702 |
| Acetone | C3 H6 O | 58.04161 | 7.656 | 75005116 |
| NP-013538 | C12 H16 O8 | 310.064 | 8.082 | 74412908 |
| 1-(3-acetyl-2,4,6-trihydroxyphenyl)ethan-1-one | C10 H10 O5 | 210.0514 | 4.967 | 73638360 |
| Acetone | C3 H6 O | 58.04161 | 8.362 | 73444706 |
| Ethyl 4-{[4-amino-6-(3,4-dihydro-2(1H)-isoquinolinyl)-1,3,5-triazin-2-yl]methyl}-1-piperazinecarboxylate | C20 H27 N7 O2 | 397.222 | 21.242 | 73250883 |
| Isobutyraldehyde | C4 H8 O | 72.05707 | 1.513 | 73170860 |
| 4-oxo-4,5,6,7-tetrahydrobenzo[b]furan-3-carboxylic acid | C9 H8 O4 | 180.041 | 4.958 | 70684231 |
| Pyrogallol | C6 H6 O3 | 126.0308 | 10.757 | 69159271 |
| Acetone | C3 H6 O | 58.04161 | 9.001 | 68442925 |
| Calcium oxide | Ca O | 55.9573 | 24.01 | 67939137 |
| 4-Methoxycinnamic acid | C10 H10 O3 | 160.0514 | 21.762 | 63574480 |
| methyl 4-[2-(aminocarbonyl)carbohydrazonoyl]-1-methyl-1H-pyrrole-2-carboxylate | C9 H12 N4 O3 | 246.0719 | 4.981 | 63258106 |
| N4-(3-chloro-4-fluorophenyl)-6-methylpyrimidine-2,4-diamine hydrochloride | C11 H10 Cl F N4 | 252.0613 | 8.693 | 62010446 |
| 4-Nitro-N-(1H-tetrazol-5-yl)benzamide | C8 H6 N6 O3 | 234.0509 | 13.761 | 60472634 |
| Acetone | C3 H6 O | 58.04161 | 7.887 | 58766116 |
| N-Methyl-2-pyrrolidone | C5 H9 N O | 99.06778 | 23.744 | 58027734 |
| NP-013538 | C12 H16 O8 | 310.064 | 8.734 | 55750127 |
| Magnesium sulfide | Mg S | 55.95689 | 24.001 | 55335236 |
| N,N'-Dimethylidyne-1,2-ethanediaminium | C4 H6 N2 | 82.05236 | 23.581 | 54909811 |
| N4-(3-chloro-4-fluorophenyl)-6-methylpyrimidine-2,4-diamine hydrochloride | C11 H10 Cl F N4 | 252.0613 | 5.675 | 53808164 |
| Methyl palmitate | C17 H34 O2 | 287.2801 | 16.049 | 52790975 |
| (1-Aminoethyl)(hydroxy)oxophosphonium | C2 H7 N O2 P | 108.0204 | 8.541 | 51850163 |
| Acetone | C3 H6 O | 58.04161 | 5.104 | 51693435 |
| Acetone | C3 H6 O | 58.04161 | 7.776 | 51247243 |
| N4-(3-chloro-4-fluorophenyl)-6-methylpyrimidine-2,4-diamine hydrochloride | C11 H10 Cl F N4 | 252.0613 | 6.129 | 51228989 |
| 5-hydroxy-4-methoxy-5,6-dihydro-2H-pyran-2-one | C6 H8 O4 | 144.0413 | 5.211 | 51007901 |
| Acetone | C3 H6 O | 58.04161 | 6.228 | 50536111 |
| 3,4-Dihydroxybenzaldehyde | C7 H6 O3 | 138.0307 | 4.932 | 49431850 |
| 2,9,10-Trioxa-6-aza-1-borabicyclo[4.3.3]dodecane | C7 H14 B N O3 | 171.107 | 21.211 | 49290107 |
| Isobutyraldehyde | C4 H8 O | 72.05707 | 3.562 | 48948367 |
| Acetamide | C2 H5 N O | 59.03684 | 23.648 | 48319336 |
| Acetone | C3 H6 O | 58.04161 | 4.98 | 46543564 |
| N-[3-(2-methyl-4-pyrimidinyl)phenyl]-1,3-benzothiazole-2-carboxamide | C19 H14 N4 O S | 346.0847 | 4.893 | 45369495 |
|  | C5 H3 N4 O P | 166.0044 | 23.992 | 44049767 |
| Acetyl chloride-d3 | C2 Cl D3 O | 81.006 | 23.998 | 43976526 |
| NP-013538 | C12 H16 O8 | 310.064 | 9.114 | 43682310 |
| 5-phenyl-2,3-dihydro-1H-1,4-benzodiazepin-2-one | C15 H12 N2 O | 258.0718 | 4.983 | 43560170 |
| (3,3-Difluoropropyl)silane | C3 H8 F2 Si | 110.036 | 4.918 | 43382209 |
| Acetone | C3 H6 O | 58.04161 | 8.504 | 43081723 |
| Acetone | C3 H6 O | 58.04161 | 5.843 | 42722229 |
| Acetone | C3 H6 O | 58.04161 | 8.183 | 42441974 |
| Eucalyptol | C10 H18 O | 136.1242 | 20.533 | 41554777 |
| 4-Methoxycinnamic acid | C10 H10 O3 | 178.0619 | 21.777 | 40432450 |
| 4-Hydroxybenzaldehyde | C7 H6 O2 | 122.0359 | 4.994 | 40379635 |
| Phosphonoacetate | C2 H4 O5 P | 138.9797 | 23.988 | 39539047 |
| N,N'-Dimethylidyne-1,2-ethanediaminium | C4 H6 N2 | 82.05234 | 23.668 | 39108316 |
| [Similar to: Isosulochrin; ΔMass: -182.0590 Da] | C2 H7 N4 O2 P | 150.0306 | 4.974 | 38913697 |
| 1'-Naphthoyl indole | C19 H13 N O | 271.0969 | 14.208 | 38266716 |
| Propionitrile | C3 H5 N | 55.04199 | 19.429 | 37310892 |
| Phthaldialdehyde | C8 H6 O2 | 134.0359 | 4.949 | 37299456 |
| Crotamiton | C13 H17 N O | 203.1296 | 19.455 | 37005629 |
| NP-022231 | C6 H13 N O4 | 185.0676 | 5.107 | 36755890 |
| Isobutyraldehyde | C4 H8 O | 72.05707 | 0.739 | 36131305 |
| Isobutyraldehyde | C4 H8 O | 72.05707 | 4.684 | 35860363 |
| Acetone | C3 H6 O | 58.04161 | 8.911 | 35502249 |
| Vinylacetylene | C4 H4 | 52.03113 | 8.85 | 35464317 |
| Propionitrile | C3 H5 N | 55.04199 | 19.82 | 35356886 |
| 1-Phenylethan-1-d1-ol | C8 H9 D O | 123.0789 | 22.424 | 35125283 |
| N4-(3-chloro-4-fluorophenyl)-6-methylpyrimidine-2,4-diamine hydrochloride | C11 H10 Cl F N4 | 252.0613 | 9.186 | 34493229 |
| Acetone | C3 H6 O | 58.04161 | 7.418 | 34098415 |
| 1-Phenylethan-1-d1-ol | C8 H9 D O | 123.0789 | 21.763 | 33880707 |
| 1-Allyl-3-[(6aS,8S)-6,12-dioxo-2-(3-thienyl)-5,6,6a,7,8,9,10,12-octahydropyrido[2,1-c][1,4]benzodiazepin-8-yl]urea | C21 H22 N4 O3 S | 432.1231 | 4.996 | 33771292 |
| Propionitrile | C3 H5 N | 55.04199 | 19.654 | 33661228 |
| Acetone | C3 H6 O | 58.04161 | 4.397 | 33616636 |
| Propionitrile | C3 H5 N | 55.04199 | 24.656 | 33173473 |
| CLOFAZIMINE | C27 H22 Cl2 N4 | 472.115 | 8.834 | 32947574 |
| butadiene | C4 H6 | 54.04677 | 5.108 | 32934649 |
| Avobenzone | C20 H22 O3 | 310.1548 | 21.573 | 32904518 |
| 2-(2-Phenylethylidene)-1,3-dithiane | C12 H14 S2 | 222.0546 | 4.64 | 32405015 |
| Acetone | C3 H6 O | 58.04161 | 8.816 | 31553891 |
| Isobutyraldehyde | C4 H8 O | 72.05707 | 1.792 | 30938103 |
| 1-Phenylethan-1-d1-ol | C8 H9 D O | 123.0789 | 22.166 | 30751829 |
| NP-011150 | C22 H24 O8 | 454.1052 | 9.267 | 30085740 |
| Acrylic acid | C3 H4 O2 | 72.02069 | 4.967 | 29980402 |
| Germylmethanetriol | C H6 Ge O3 | 139.9535 | 4.378 | 29946817 |
| Acetone | C3 H6 O | 58.04161 | 4.874 | 28890614 |
| Eucalyptol | C10 H18 O | 136.1242 | 8.397 | 28743579 |
| Pyrogallol | C6 H6 O3 | 126.0308 | 10.502 | 27070806 |
| Acetone | C3 H6 O | 58.04161 | 12.15 | 26888067 |
| Difluoro(thioxo)phosphonium | F2 P S | 100.9418 | 24.026 | 26830043 |
| Eucalyptol | C10 H18 O | 136.1242 | 24.209 | 26743070 |
| Calcium oxide | Ca O | 55.95729 | 4.371 | 26649207 |
| 2,9,10-Trioxa-6-aza-1-borabicyclo[4.3.3]dodecane | C7 H14 B N O3 | 171.107 | 23.651 | 26174465 |
| Isobutyraldehyde | C4 H8 O | 72.05707 | 7.8 | 25783811 |
| NP-008999 | C22 H28 O10 | 490.125 | 5.835 | 25632096 |
| 2-(benzylamino)-1,3-thiazole-5-carboxylic acid | C11 H10 N2 O2 S | 234.0511 | 8.711 | 25203709 |
| Acetone | C3 H6 O | 58.04161 | 2.789 | 25034561 |
| Eucalyptol | C10 H18 O | 136.1242 | 24.501 | 24970528 |
| Acetamide | C2 H5 N O | 59.03684 | 20.331 | 24927462 |
| 2-(2-Phenylethylidene)-1,3-dithiane | C12 H14 S2 | 222.0547 | 7.787 | 23330508 |
|  | C10 H18 N10 | 278.1713 | 20.151 | 23314616 |
| (1-Aminoethyl)(hydroxy)oxophosphonium | C2 H7 N O2 P | 108.0204 | 13.746 | 23160567 |
| N,N'-Dimethylidyne-1,2-ethanediaminium | C4 H6 N2 | 82.05235 | 22.31 | 23052267 |
| Acetone | C3 H6 O | 58.04161 | 5.661 | 23026560 |
| NP-011150 | C22 H24 O8 | 454.1052 | 9.163 | 22863713 |
| 4,6,8-trimethylquinoline-2-thiol | C12 H13 N S | 203.0778 | 4.983 | 22825354 |
| Propionitrile | C3 H5 N | 55.04199 | 16.416 | 22744015 |
| Propionitrile | C3 H5 N | 55.04199 | 24.754 | 22740003 |
| 5-Methoxysalicylic acid | C8 H8 O4 | 168.0411 | 4.896 | 22719346 |
| Eucalyptol | C10 H18 O | 136.1242 | 19.308 | 22698979 |
| 1-Phenylethan-1-d1-ol | C8 H9 D O | 123.0789 | 23.366 | 22667926 |
| NP-011150 | C22 H24 O8 | 454.1051 | 9.387 | 22497735 |
| Propionitrile | C3 H5 N | 55.04199 | 12.784 | 22408245 |
| Propionitrile | C3 H5 N | 55.04199 | 19.178 | 21729684 |
| Propionitrile | C3 H5 N | 55.04199 | 19.258 | 21604529 |
| (Ethylimino)di-2,1-ethanediyl dicarbamimidothioate | C8 H19 N5 S2 | 249.1093 | 18.046 | 21602250 |
| N,N'-Dimethylidyne-1,2-ethanediaminium | C4 H6 N2 | 82.05237 | 22.898 | 21596505 |
| Vinylacetylene | C4 H4 | 52.03113 | 4.815 | 21547343 |
| Ethyl 4-{[4-amino-6-(3,4-dihydro-2(1H)-isoquinolinyl)-1,3,5-triazin-2-yl]methyl}-1-piperazinecarboxylate | C20 H27 N7 O2 | 397.222 | 21.404 | 21506493 |
| Propionitrile | C3 H5 N | 55.04199 | 16.269 | 21148045 |
| NP-011150 | C22 H24 O8 | 454.1052 | 9.601 | 20760219 |
| Phosphorimidic triamide | H7 N4 P | 94.04125 | 4.978 | 20519662 |
| 3,5-di-tert-Butyl-4-hydroxybenzaldehyde | C15 H22 O2 | 234.1603 | 19.455 | 20397520 |
| Eucalyptol | C10 H18 O | 136.1242 | 7.736 | 20384258 |
|  |  |  |  |  |

**Table S2.** LC/MS/MS Orbitrap analysis of intermediate substances from carbon dots synthesis from 5%w/v sucrose solution at 280 °C using 1 mL min^-1^ flow rate in a continuous flow reactor system (Top compounds with most area ≥ 2×10^7^)

| Name | Formula | Molecular Weight | RT [min] | Area (Max.) |
| --- | --- | --- | --- | --- |
| 5-Hydroxymethyl-2-furaldehyde | C6 H6 O3 | 126.0311 | 8.862 | 46631985529 |
| Pyridine | C5 H5 N | 79.04191 | 22.111 | 11580196713 |
| Cyclopentadienone | C5 H4 O | 80.02591 | 8.877 | 4070862647 |
| Pyrone | C5 H4 O2 | 96.02065 | 10.292 | 3580569146 |
| (2S)-3,3-Dichloro-1,2-propanediol | C3 H6 Cl2 O2 | 143.9743 | 22.036 | 2821290744 |
| 1,2-Dioxin | C4 H4 O2 | 84.02081 | 4.951 | 2626111357 |
| 1,2-Benzoquinone | C6 H4 O2 | 108.0206 | 9.926 | 2018362065 |
| Acetamide | C2 H5 N O | 59.03703 | 21.175 | 1978100486 |
| 5-Hydroxymethyl-2-furaldehyde | C6 H6 O3 | 126.031 | 9.561 | 1869314690 |
| Propionitrile | C3 H5 N | 55.04214 | 19.308 | 1846297166 |
| Diethylamine | C4 H11 N | 73.08891 | 21.566 | 1794184247 |
| Mosher's acid | C10 H9 F3 O3 | 234.0514 | 13.751 | 1707753528 |
| 5-Hydroxymethyl-2-furaldehyde | C6 H6 O3 | 126.031 | 5.089 | 1457241790 |
| (2E)-5-Oxo-2,4-pentadienal | C5 H4 O2 | 96.02065 | 5.072 | 1369296092 |
| Propionitrile | C3 H5 N | 55.04214 | 16.331 | 1353708176 |
| 1-Cyano-3-butyne | C5 H5 N | 79.04191 | 17.421 | 1283347712 |
| 1,2-Benzoquinone | C6 H4 O2 | 108.0206 | 9.624 | 1212239457 |
| (2E)-5-Oxo-2,4-pentadienal | C5 H4 O2 | 96.02065 | 5.708 | 1087127933 |
| 1,2-Benzoquinone | C6 H4 O2 | 108.0206 | 13.754 | 850962539.9 |
| Acetamide | C2 H5 N O | 59.03703 | 23.341 | 771823540.7 |
| 5-Hydroxymethyl-2-furaldehyde | C6 H6 O3 | 126.0311 | 4.835 | 723421401.5 |
| 5-Phenoxy-2-furoic acid | C11 H8 O4 | 204.0413 | 13.633 | 662697450.6 |
| Cyclopentadienone | C5 H4 O | 80.02591 | 9.919 | 662501181.4 |
| Propionitrile | C3 H5 N | 55.04214 | 17.85 | 640690279.7 |
| catecholate | C6 H4 O2 | 108.0206 | 5.051 | 638106368.8 |
| Cyclopentadienone | C5 H4 O | 80.02591 | 9.375 | 610381950.7 |
| Ethyl propiolate | C5 H6 O2 | 98.03641 | 7.736 | 580067565.3 |
| Cyclopentadienone | C5 H4 O | 80.02591 | 13.753 | 549977582.1 |
| Furan | C4 H4 O | 68.02599 | 4.838 | 510540557.3 |
| lactide | C6 H8 O4 | 144.0416 | 7.696 | 494761838.8 |
| 5-Hydroxymethyl-2-furaldehyde | C6 H6 O3 | 126.031 | 10.173 | 479787928.5 |
| 1,2-Dioxin | C4 H4 O2 | 84.02081 | 6.211 | 458748005.5 |
| Acetone | C3 H6 O | 58.04171 | 7.977 | 452477043.9 |
| Cyclopentadienone | C5 H4 O | 80.02591 | 11.229 | 451037139.9 |
| MFCD00010043 | C16 H10 S | 234.0515 | 11.354 | 426441377.2 |
| Pyrogallol | C6 H6 O3 | 126.031 | 10.448 | 373897816.7 |
| Sulfamic acid | H3 N O3 S | 96.98364 | 24.005 | 366193056.6 |
| Phloroglucinol | C6 H6 O3 | 126.0311 | 11.549 | 309099410.6 |
| 1,2-Benzoquinone | C6 H4 O2 | 108.0206 | 10.927 | 302546992 |
| Cyclopentadienone | C5 H4 O | 80.02591 | 5.517 | 299218673.5 |
| Scoparone | C11 H10 O4 | 206.0569 | 13.753 | 287436364 |
| 1,4-Pentadiyn-3-one | C5 H2 O | 78.0104 | 24.008 | 284263199.5 |
| NP-013538 | C12 H16 O8 | 310.0647 | 8.365 | 274593709 |
| muconaldehyde | C6 H6 O2 | 110.0363 | 12.525 | 264383427.6 |
| Pyrogallol | C6 H6 O3 | 126.031 | 10.722 | 254418222.5 |
| Cyclopentadienone | C5 H4 O | 80.02591 | 9.663 | 253570750.8 |
| Furfuranol | C5 H6 O2 | 98.03644 | 5.074 | 252795159.2 |
| 4-KETO-2-PENTENAL | C5 H6 O2 | 98.03641 | 9.211 | 247657251.9 |
| D-(+)-Maltose | C12 H22 O11 | 364.0958 | 4.732 | 244444310.4 |
| Propionitrile | C3 H5 N | 55.04214 | 12.766 | 238696212.8 |
| N4-(3-chloro-4-fluorophenyl)-6-methylpyrimidine-2,4-diamine hydrochloride | C11 H10 Cl F N4 | 252.062 | 8.62 | 235684894.8 |
| 2-[3-methyl-2-(methylimino)-4-oxo-1,3-thiazolan-5-yl]acetic acid | C7 H10 N2 O3 S | 202.0445 | 4.716 | 235075238.7 |
| Propionitrile | C3 H5 N | 55.04214 | 11.412 | 234949049.5 |
| Furfuranol | C5 H6 O2 | 98.03645 | 4.83 | 231583957.4 |
| catecholate | C6 H4 O2 | 108.0206 | 8.392 | 222696142 |
| N4-(3-chloro-4-fluorophenyl)-6-methylpyrimidine-2,4-diamine hydrochloride | C11 H10 Cl F N4 | 252.0621 | 9.616 | 222408592.9 |
| (Nitroimino)dimethanol | C2 H6 N2 O4 | 122.0324 | 23.99 | 222195514.6 |
| N-[3-(2-methyl-4-pyrimidinyl)phenyl]-1,3-benzothiazole-2-carboxamide | C19 H14 N4 O S | 346.0856 | 5.128 | 218459543.7 |
| N4-(3-chloro-4-fluorophenyl)-6-methylpyrimidine-2,4-diamine hydrochloride | C11 H10 Cl F N4 | 252.062 | 9.25 | 216592307.2 |
| Furan | C4 H4 O | 68.02599 | 5.326 | 209128002.2 |
| 5-hydroxy-4-methoxy-5,6-dihydro-2H-pyran-2-one | C6 H8 O4 | 144.0416 | 4.83 | 204836948.7 |
| Pyrogallol | C6 H6 O3 | 126.031 | 11.187 | 194056909.6 |
| (Z)-1-cyano-1,3-butadiene | C5 H5 N | 79.04191 | 12.714 | 186639636.7 |
| Herniarin | C10 H8 O3 | 176.0465 | 13.742 | 182965173.2 |
| Acetamide | C2 H5 N O | 59.03703 | 16.041 | 182768103.9 |
| Furan | C4 H4 O | 68.02599 | 5.08 | 176041557.6 |
| N4-(3-chloro-4-fluorophenyl)-6-methylpyrimidine-2,4-diamine hydrochloride | C11 H10 Cl F N4 | 252.062 | 11.675 | 169148356.6 |
| Propionitrile | C3 H5 N | 55.04214 | 10.846 | 165472150.2 |
| N4-(3-chloro-4-fluorophenyl)-6-methylpyrimidine-2,4-diamine hydrochloride | C11 H10 Cl F N4 | 252.062 | 5.021 | 165297156.8 |
| 3-hydroxy-3-methylpentanedioic acid | C6 H10 O5 | 144.0416 | 5.365 | 163262315.2 |
| N4-(3-chloro-4-fluorophenyl)-6-methylpyrimidine-2,4-diamine hydrochloride | C11 H10 Cl F N4 | 252.062 | 9.958 | 159308903.6 |
| Pyrogallol | C6 H6 O3 | 126.031 | 10.952 | 158513634.8 |
| 3-hydroxy-3-methylpentanedioic acid | C6 H10 O5 | 144.0416 | 5.742 | 152657383.6 |
| (2E)-5-Oxo-2,4-pentadienal | C5 H4 O2 | 96.02065 | 11.343 | 146993030 |
| NP-013538 | C12 H16 O8 | 310.0647 | 7.781 | 143815911.6 |
| 3-[3-(3-Methoxypropoxy)propoxy]propyl acrylate | C13 H24 O5 | 260.1614 | 20.13 | 137606154.1 |
| Propionitrile | C3 H5 N | 55.04214 | 10.184 | 128790278.8 |
| Vinylacetylene | C4 H4 | 52.03124 | 8.869 | 126700905.9 |
| Hexa-2,4-dienedial | C6 H6 O2 | 110.0363 | 5.393 | 121519599.5 |
| Acetamide | C2 H5 N O | 59.03703 | 12.236 | 118806412 |
| Cyclopentadienone | C5 H4 O | 80.02591 | 8.396 | 111889603.9 |
| Methyl palmitate | C17 H34 O2 | 287.2808 | 16.389 | 107881903.7 |
| Benzothiazole | C7 H5 N S | 135.0137 | 20.663 | 106872173 |
| [Similar to: 3-hydroxy-N-(1-hydroxy-4-methylpentan-2-yl)-5-oxo-6-phenylhexanamide; ΔMass: 2.9098 Da] | C8 H16 N6 O8 | 324.1038 | 4.825 | 106655120.8 |
| NP-022075 | C23 H22 O7 | 432.124 | 9.15 | 104808113 |
| Cyclopentadienone | C5 H4 O | 80.02591 | 7.085 | 102313862.4 |
| 3-hydroxy-3-methylpentanedioic acid | C6 H10 O5 | 184.034 | 5.094 | 99217362.04 |
| 1,4-Cyclohexadiene | C6 H8 | 80.06223 | 24.193 | 99078062.09 |
| [Similar to: NP-018314; ΔMass: -235.2570 Da] | C8 H18 N5 O4 P | 279.1088 | 13.755 | 96369182.79 |
| Pyrogallol | C6 H6 O3 | 126.031 | 0.752 | 95798577.54 |
| Eucalyptol | C10 H18 O | 136.1245 | 24.304 | 95567206.86 |
| Propionitrile | C3 H5 N | 55.04214 | 24.05 | 95480606.58 |
| Acetone | C3 H6 O | 58.04171 | 7.754 | 93818021.91 |
| Propionitrile | C3 H5 N | 55.04214 | 13.153 | 92637549.86 |
| 3-hydroxy-3-methylpentanedioic acid | C6 H10 O5 | 144.0416 | 6.268 | 90769089.93 |
| 3-hydroxy-3-methylpentanedioic acid | C6 H10 O5 | 144.0416 | 5.102 | 87203527.05 |
| NP-013538 | C12 H16 O8 | 310.0647 | 8.914 | 85870493.1 |
| 4-KETO-2-PENTENAL | C5 H6 O2 | 98.03641 | 8.854 | 85242164.96 |
| 2,7,9-Trimethyl-4H-pyrido[3',2':4,5]thieno[3,2-d][1,3]oxazin-4-one | C12 H10 N2 O2 S | 246.0491 | 13.257 | 84878171.4 |
| 1,2-Benzoquinone | C6 H4 O2 | 108.0206 | 6.988 | 82168274.06 |
| N4-(3-chloro-4-fluorophenyl)-6-methylpyrimidine-2,4-diamine hydrochloride | C11 H10 Cl F N4 | 252.062 | 6.929 | 82024663.4 |
| Propionitrile | C3 H5 N | 55.04214 | 9.581 | 80643473.36 |
| 1,2-Benzoquinone | C6 H4 O2 | 108.0206 | 13.263 | 76796351.47 |
| Furan | C4 H4 O | 68.02599 | 10.29 | 76678669.57 |
| N4-(3-chloro-4-fluorophenyl)-6-methylpyrimidine-2,4-diamine hydrochloride | C11 H10 Cl F N4 | 252.062 | 10.908 | 74793956.87 |
| 2,3-Dihydroxypropyl 5-oxoprolinate | C8 H13 N O5 | 203.0785 | 5.134 | 73392543.25 |
| N4-(3-chloro-4-fluorophenyl)-6-methylpyrimidine-2,4-diamine hydrochloride | C11 H10 Cl F N4 | 252.062 | 10.497 | 71338592.23 |
| Pyrone | C5 H4 O2 | 96.02065 | 10.886 | 71237120.22 |
| Eucalyptol | C10 H18 O | 136.1245 | 13.742 | 71056586.28 |
| Acetone | C3 H6 O | 58.04171 | 9.171 | 69955740.4 |
| (3R,4S)-4,6,8-Trihydroxy-7-methoxy-3-methyl-3,4-dihydro-1H-isochromen-1-one | C11 H12 O6 | 240.0623 | 4.983 | 68892391.52 |
| Acetone | C3 H6 O | 58.04171 | 9.694 | 68888740.3 |
| 2-(3,4-dihydroxyphenyl)acetamide | C8 H9 N O3 | 167.0574 | 8.845 | 68379875.2 |
| N4-(3-chloro-4-fluorophenyl)-6-methylpyrimidine-2,4-diamine hydrochloride | C11 H10 Cl F N4 | 252.062 | 10.223 | 68259975.59 |
| Pyrogallol | C6 H6 O3 | 126.031 | 11.321 | 68131468.72 |
| Calcium oxide | Ca O | 55.95745 | 24.016 | 67884194.75 |
| Calcium oxide | Ca O | 55.95744 | 4.376 | 67355515.99 |
| Pyrone | C5 H4 O2 | 96.02065 | 10.632 | 67223663.78 |
|  | C8 H16 N2 P2 S | 234.0515 | 8.664 | 66948682.76 |
| 3-hydroxy-3-methylpentanedioic acid | C6 H10 O5 | 144.0416 | 8.638 | 66930033.96 |
| Biochanin A | C16 H12 O5 | 284.0668 | 16.323 | 66729641.66 |
| N4-(3-chloro-4-fluorophenyl)-6-methylpyrimidine-2,4-diamine hydrochloride | C11 H10 Cl F N4 | 252.062 | 6.1 | 66022342.25 |
| Furan | C4 H4 O | 68.02599 | 5.696 | 65837898.48 |
| (2E)-3-(3,4-dimethoxyphenyl)prop-2-enoic acid | C11 H12 O4 | 190.0622 | 16.825 | 65712099.32 |
| Ethyl (3S)-3-cyano-3-hydroxypropanoate | C6 H9 N O3 | 143.0576 | 4.927 | 65381533.93 |
| butadiene | C4 H6 | 54.04688 | 5.089 | 64411703.14 |
| 4-Methylumbelliferone | C10 H8 O3 | 176.0465 | 15.923 | 64177055.5 |
| Scoparone | C11 H10 O4 | 206.057 | 12.729 | 63299038.9 |
| NP-011150 | C22 H24 O8 | 454.1059 | 9.817 | 63289966.45 |
| Phosphoramidisocyanatidic chloride | C H2 Cl N2 O2 P | 139.9538 | 4.381 | 63051038.02 |
| Acetone | C3 H6 O | 58.04171 | 4.718 | 61707190.42 |
| Acetamide | C2 H5 N O | 59.03703 | 24.531 | 61331216.73 |
| N-(cyclopropylmethyl)-N'-phenylurea | C11 H14 N2 O | 228.0622 | 4.964 | 60752032.54 |
| Betaine | C5 H11 N O2 | 117.0785 | 4.815 | 60525724.69 |
| 1,4-Cyclohexadiene | C6 H8 | 80.06223 | 20.584 | 60247115.63 |
| 1,4-Cyclohexadiene | C6 H8 | 80.06223 | 13.669 | 60155538.94 |
|  | C8 H19 N7 O8 | 341.1301 | 4.999 | 60127543.34 |
| Acetone | C3 H6 O | 58.04171 | 5.108 | 59948423.71 |
| NP-007735 | C18 H24 O12 | 432.124 | 9.467 | 59503717.77 |
| NP-013538 | C12 H16 O8 | 310.0647 | 8.701 | 59315853.84 |
| Pyrogallol | C6 H6 O3 | 126.031 | 11.792 | 59238281.58 |
| N-acetyl-L-2-aminoadipic acid | C8 H13 N O5 | 203.0784 | 4.824 | 58325630.83 |
| Scoparone | C11 H10 O4 | 206.0569 | 9.854 | 57138783.2 |
| Furan | C4 H4 O | 68.02599 | 5.988 | 55819805.21 |
|  | C10 H19 N7 O6 | 333.1406 | 8.414 | 54156009.01 |
| Acetone | C3 H6 O | 58.04171 | 9.456 | 53908595.62 |
| Acetone | C3 H6 O | 58.04171 | 5.23 | 53198197.88 |
| 1H-indol-3-yl(pyridin-2-yl)methanol | C14 H12 N2 O | 246.0726 | 4.981 | 51874918.31 |
| Acetone | C3 H6 O | 58.04171 | 11.986 | 51719130.38 |
| α-Lactose | C12 H22 O11 | 342.1141 | 4.754 | 50021654.67 |
| N-Methyl-2-pyrrolidone | C5 H9 N O | 99.06808 | 23.869 | 49951065.22 |
| Acetone | C3 H6 O | 58.04171 | 8.334 | 49783881.42 |
| 3-Hydroxy-1,2-benzoquinone | C6 H4 O3 | 124.0156 | 8.915 | 49181487.45 |
| Ethyl 4-{[4-amino-6-(3,4-dihydro-2(1H)-isoquinolinyl)-1,3,5-triazin-2-yl]methyl}-1-piperazinecarboxylate | C20 H27 N7 O2 | 397.2231 | 21.255 | 48566846.77 |
| Cyclopentadienone | C5 H4 O | 80.02591 | 12.851 | 48463540.68 |
|  | C10 H8 N6 O5 | 292.0543 | 9.983 | 47850556.75 |
| butadiene | C4 H6 | 54.04688 | 13.746 | 47481300.14 |
| Pyrone | C5 H4 O2 | 96.02065 | 9.452 | 46353067.12 |
| Cyclopentadienone | C5 H4 O | 80.02591 | 13.257 | 46282336.55 |
| Acetone | C3 H6 O | 58.04171 | 11.3 | 45920977.58 |
| Acetone | C3 H6 O | 58.04171 | 8.718 | 45817351.66 |
| Methyl 4-oxo-2-butenoate | C5 H6 O3 | 114.0312 | 4.989 | 45635511.77 |
| Acetamide | C2 H5 N O | 59.03703 | 23.949 | 45272787.29 |
| Scoparone | C11 H10 O4 | 206.0569 | 11.357 | 44953819.81 |
| 1-Thia-5-azaspiro[5.5]undecane | C9 H17 N S | 171.1074 | 21.227 | 44953465.75 |
| 4-KETO-2-PENTENAL | C5 H6 O2 | 98.03641 | 10.127 | 44609147.09 |
| Acetone | C3 H6 O | 58.04171 | 5.822 | 44490602.71 |
| Eucalyptol | C10 H18 O | 136.1245 | 20.492 | 44035657.77 |
| Acetone | C3 H6 O | 58.04171 | 9.835 | 43580418.06 |
| 2-(2-Pyrazinyl)ethanamine | C6 H9 N3 | 123.0792 | 23.738 | 43525984.2 |
| 2-(2-Pyrazinyl)ethanamine | C6 H9 N3 | 123.0792 | 23.856 | 42282260.57 |
| 1-[2,6-dichloro-4-(trifluoromethyl)phenyl]-3-methyl-1H-pyrazole-4,5-dione 4-(N-phenylhydrazone) | C17 H11 Cl2 F3 N4 O | 414.0333 | 21.202 | 42127285.05 |
| Acetone | C3 H6 O | 58.04171 | 8.647 | 41932763.05 |
| Crotamiton | C13 H17 N O | 203.1301 | 19.457 | 41832789.78 |
| CLOFAZIMINE | C27 H22 Cl2 N4 | 472.1166 | 8.857 | 41779465.2 |
| Aflatoxin B2 | C17 H14 O6 | 336.0593 | 14.327 | 41155852.75 |
| neosaxitoxin | C10 H17 N7 O5 | 315.1298 | 9.653 | 41131217.39 |
| 1,4-Cyclohexadiene | C6 H8 | 80.06223 | 24.572 | 39419527.44 |
| N4-(3-chloro-4-fluorophenyl)-6-methylpyrimidine-2,4-diamine hydrochloride | C11 H10 Cl F N4 | 252.062 | 11.415 | 39361433.41 |
| Acetone | C3 H6 O | 58.04171 | 8.387 | 38844989.39 |
| 4-[5-(4-chloro-2-nitrophenyl)-2-furyl]-2,6-dimethyl-1,4-dihydropyridine-3,5-dicarbonitrile | C19 H13 Cl N4 O3 | 380.0698 | 4.722 | 37490809.33 |
| Scoparone | C11 H10 O4 | 206.0569 | 8.714 | 37360460.62 |
| 3-(2-Chloroethyl)-2-methyl-4H-pyrido(1,2-a)pyrimidin-4-one | C11 H11 Cl N2 O | 222.0552 | 7.941 | 36716587.53 |
| 2-Hydrazino-2-thioxoacetamide | C2 H5 N3 O S | 119.0158 | 4.314 | 35925636.58 |
| Ethyl N-(1,4-thiazinan-4-ylcarbothioyl)carbamate | C8 H14 N2 O2 S2 | 234.0515 | 9.945 | 35668093.87 |
| 1-(4-hydroxyphenyl)propane-1,2-diol | C9 H12 O3 | 190.0622 | 16.189 | 35529660.53 |
| 3,5-Bis{[bis(2-methyl-2-propanyl)phosphino]methyl}-2,4,6-trimethylphenol | C27 H50 O P2 | 452.3336 | 11.993 | 35447302.99 |
| Furan | C4 H4 O | 68.02599 | 6.445 | 35050900.53 |
| Scoparone | C11 H10 O4 | 206.0569 | 9.235 | 34520729.78 |
| butadiene | C4 H6 | 54.04688 | 5.707 | 34408609.02 |
| 1-(3-acetyl-2,4,6-trihydroxyphenyl)ethan-1-one | C10 H10 O5 | 210.052 | 4.962 | 33985890.77 |
| 1'-Naphthoyl indole | C19 H13 N O | 271.0976 | 14.209 | 33632403.63 |
| 1,4-Cyclohexadiene | C6 H8 | 80.06223 | 18.564 | 32269117.25 |
| 4-KETO-2-PENTENAL | C5 H6 O2 | 98.03641 | 8.612 | 31766834.76 |
| N1-[2-(2-hydroxy-3-{[5-(trifluoromethyl)-2-pyridyl]sulfonyl}propoxy)phenyl]acetamide | C17 H17 F3 N2 O5 S | 418.0853 | 11.53 | 31685588.43 |
| 1,2-Benzoquinone | C6 H4 O2 | 108.0206 | 16.814 | 30610340.02 |
| 3-(2-Chloroethyl)-2-methyl-4H-pyrido(1,2-a)pyrimidin-4-one | C11 H11 Cl N2 O | 222.0552 | 8.388 | 30397972.14 |
| N4-(3-chloro-4-fluorophenyl)-6-methylpyrimidine-2,4-diamine hydrochloride | C11 H10 Cl F N4 | 252.062 | 6.493 | 28878342.45 |
| (3E)-4-methyl-3-(phenylmethylidene)-2,3,4,5-tetrahydro-1H-1,4-benzodiazepine-2,5-dione | C17 H14 N2 O2 | 300.0804 | 6.478 | 28718059.99 |
| 5-Hydroxy-4-pentyn-2-one | C5 H6 O2 | 98.03641 | 9.769 | 27998909.76 |
| Eucalyptol | C10 H18 O | 136.1245 | 8.818 | 27668097.99 |
| Phosphonoacetate | C2 H4 O5 P | 138.9801 | 24.006 | 27502541.49 |
| Pyrogallol | C6 H6 O3 | 126.0311 | 11.987 | 26594529.14 |
| Acetone | C3 H6 O | 58.04171 | 2.725 | 26584247.56 |
| (p-Hydroxyphenyl)glyoxal | C8 H6 O3 | 150.031 | 11.178 | 26356996.39 |
| neosaxitoxin | C10 H17 N7 O5 | 315.1298 | 9.584 | 26350162.36 |
| 2-(2-Pyrazinyl)ethanamine | C6 H9 N3 | 123.0792 | 21.461 | 26253617.85 |
| 4-KETO-2-PENTENAL | C5 H6 O2 | 98.03641 | 9.568 | 25378350.27 |
| 2-Furoic acid | C5 H4 O3 | 112.0156 | 5.24 | 24241079.58 |
| Ethyl 4-{[4-amino-6-(3,4-dihydro-2(1H)-isoquinolinyl)-1,3,5-triazin-2-yl]methyl}-1-piperazinecarboxylate | C20 H27 N7 O2 | 397.2231 | 21.602 | 23883691.04 |
| Pyrogallol | C6 H6 O3 | 126.0309 | 5.671 | 23728570.38 |
| Propionitrile | C3 H5 N | 55.04214 | 12.465 | 23508411.18 |
| 1,4-Cyclohexadiene | C6 H8 | 80.06223 | 8.797 | 23483495.9 |
| 3,4-Dihydroxybenzaldehyde | C7 H6 O3 | 138.0311 | 4.88 | 22942733.26 |
| [Similar to: 3-hydroxy-N-(1-hydroxy-4-methylpentan-2-yl)-5-oxo-6-phenylhexanamide; ΔMass: 2.9098 Da] | C9 H12 N10 O4 | 324.1038 | 4.914 | 22560392.8 |
| (2E)-3-(4-methoxy-2-{[(2S,3R,4S,5S,6R)-3,4,5-trihydroxy-6-(hydroxymethyl)oxan-2-yl]oxy}phenyl)prop-2-enoic acid | C16 H20 O9 | 378.0926 | 10.23 | 22424379.54 |
| Ethyl N-(1,4-thiazinan-4-ylcarbothioyl)carbamate | C8 H14 N2 O2 S2 | 234.0515 | 10.201 | 22403796.66 |
| N,N'-Dimethyl-P-phenylphosphonic dihydrazide | C8 H15 N4 O P | 214.0981 | 8.843 | 22372249.35 |
| 2-(3-Fluorophenyl)-1-(2-thienyl)ethanamine | C12 H12 F N S | 221.0677 | 13.596 | 22215854.35 |
| 4-Methoxycinnamic acid | C10 H10 O3 | 160.0518 | 21.78 | 22007848.4 |
| 4-oxo-4,5,6,7-tetrahydrobenzo[b]furan-3-carboxylic acid | C9 H8 O4 | 180.0415 | 4.949 | 21699382.3 |
| N-[3-(2-methyl-4-pyrimidinyl)phenyl]-1,3-benzothiazole-2-carboxamide | C19 H14 N4 O S | 346.0856 | 4.775 | 21695241.49 |
| NP-011150 | C22 H24 O8 | 454.1059 | 9.025 | 21674825.12 |
| Eucalyptol | C10 H18 O | 136.1245 | 12.748 | 21635192.75 |
| 4-KETO-2-PENTENAL | C5 H6 O2 | 98.03641 | 9.018 | 21600635.54 |
| Propionitrile | C3 H5 N | 55.04214 | 9.901 | 21417627.08 |
| 2-(2-Thienyl)-1,4-dihydroquinazolin-4-one | C12 H8 N2 O S | 228.0386 | 12.717 | 21270172.22 |
| Phloroglucinol | C6 H6 O3 | 126.031 | 12.207 | 21084193.98 |
| 1-(8-Methyl-4,5-dihydronaphtho[1,2-b]thiophen-2-yl)ethanone | C15 H14 O S | 242.0776 | 9.45 | 20984796.24 |
| 4-Hydroxybenzaldehyde | C7 H6 O2 | 122.0363 | 4.91 | 20610600.54 |
| Difluoro(thioxo)phosphonium | F2 P S | 100.9421 | 24.03 | 20325576.17 |
| Scoparone | C11 H10 O4 | 206.0568 | 13.116 | 20213845.72 |
| 1,4-Cyclohexadiene | C6 H8 | 80.06223 | 18.958 | 20175255.83 |
